# New Perspectives in the Assessment of Severity Scoring in Acute Pancreatitis: Development and Internal Validation of a Novel Bedside Prognostic Score (ProbScore2)

**DOI:** 10.3390/medicina62091630

**Published:** 2026-08-25

**Authors:** Zsombor Szász, Imola Török, Simona Maria Bățagă

**Affiliations:** 1 Doctoral School of Medicine and Pharmacy, George Emil Palade University of Medicine, Pharmacy, Science, and Technology of Târgu Mureș, 540139 Târgu Mureș, Romania; szaszzsombi3@gmail.com; 2 Discipline of Internal Medicine 1, M3 Department, George Emil Palade University of Medicine, Pharmacy, Science, and Technology of Târgu Mureș, 540139 Târgu Mureș, Romania; simona.bataga@umfst.ro

**Keywords:** acute pancreatitis, severity score, prognostic model, logistic regression, ROC analysis, bedside risk stratification

## Abstract

*Background and Objectives:* Existing severity scores for acute pancreatitis (AP), such as the Ranson, Glasgow-Imrie, APACHE II, BISAP, Balthazar scores, are limited by complexity, delayed availability, or insufficient discriminative accuracy. We aimed to develop a new prognostic score using parameters available at admission in any primary or regional care setting, suitable for early risk stratification and clinical decision-making in AP. *Materials and Methods:* We conducted a retrospective observational study of 194 patients admitted with acute pancreatitis, classified as mild (MILD, *n* = 59), moderately severe (*n* = 82) and severe (*n*= 53) course, according to the Revised Atlanta Classification. Candidate predictors—including demographic, clinical, inflammatory, nutritional, and radiological variables—were screened using univariable analysis, and independent predictors were identified by backward stepwise logistic regression. Discriminative performance was assessed using receiver operating characteristic (ROC) curve analysis and compared with six established severity scores (Ranson, Glasgow-Imrie, Balthazar, CTSI, BISAP, and HAPS). APACHE II was not included in this comparison because some of its required physiological variables were not consistently available outside the intensive care unit. *Results:* MODSEV (moderately severe/severe) patients showed significantly higher inflammatory, nutritional, and metabolic derangement at admission than MILD patients. Six variables—admission white blood cell count, blood urea nitrogen (BUN), pleural effusion, admission blood glucose level, platelet-to-lymphocyte ratio (PLR), and serum albumin—were retained in the final logistic regression model (ProbScore2), which demonstrated excellent discriminative ability (AUC = 0.873; 95% CI: 0.819–0.927), showing, within this retrospective cohort, a numerically higher AUC than the Glasgow-Imrie (AUC = 0.832), CTSI (0.775), Balthazar (0.758), BISAP (0.725), Ranson (0.676) and HAPS (0.574). A simplified, unweighted 0–6 point bedside version of the score retained most of this discriminative ability (AUC = 0.852), with an optimal cut-off of ≥3 points yielding a sensitivity of 77.0% and a specificity of 79.7%. *Conclusions:* ProbScore2, a novel six-variable prognostic score, demonstrated excellent discriminative performance for the early identification of moderately severe/severe acute pancreatitis, with a numerically higher AUC than established severity scoring systems evaluated within this same retrospective, single-center cohort (excluding APACHE II, which could not be calculated for all patients). These comparative findings require confirmation through prospective external validation before any conclusions about generalizable superiority can be drawn. Five of the six components are derived from routine admission blood tests, and the sixth (pleural effusion) requires only a plain chest radiograph rather than cross-sectional imaging. The score and its simplified bedside adaptation can therefore be calculated within hours of admission using resources available even in hospitals without intensive care or advanced, CT-based imaging capabilities. ProbScore2 is not yet ready for routine clinical use; prospective external validation in independent, multicenter cohorts is required before clinical implementation.

## 1. Introduction

Acute pancreatitis is one of the most common gastrointestinal conditions requiring hospital admission and represents a substantial global burden on healthcare systems. In the United States, more than 275,000 cases are recorded annually. According to the 2019 Global Burden of Disease Study, approximately 2.8 million cases of acute pancreatitis occur worldwide each year. In Romania, this corresponds to more than 14,000 cases per year, or 50.8 cases per 100,000 person-years [1,2].

The clinical course can range from mild, self-limiting inflammation to severe disease characterized by necrosis, systemic inflammatory response syndrome (SIRS), and multiple organ failure [3].

The average mortality rate reported in modern studies ranges from 1% to 5%, but this can rise to as much as 30–40% in cases complicated by necrosis and multiple organ failure requiring intensive care [4,5]. According to a population-based Swedish cohort study, acute pancreatitis is associated with an increased risk of long-term mortality even after excluding comorbidities such as malignancies and recurrent or chronic pancreatitis. Mortality was highest immediately after hospital discharge, declined during the first year, but remained elevated even beyond 10 years of follow-up [6].

Numerous scoring systems are currently available for the early assessment of acute pancreatitis severity, including the Ranson score, Glasgow-Imrie score, APACHE II, BISAP, Balthazar grade, CT Severity Index (CTSI), and modified CTSI (mCTSI); however, most of these are complex, insufficiently accurate, or rely on parameters that are unavailable in the emergency setting or cannot be evaluated until 48 h after admission [7]. The Ranson score, for example, requires 11 parameters assessed across the first 48 h and therefore cannot be finalized until two days into the admission [7,8,9]. The Glasgow-Imrie score reduces this to nine parameters, incorporating serum albumin as a marker of the capillary leak that accompanies systemic inflammation in AP, but remains fully assessable only at 48 h [10]. APACHE II, developed for the general intensive care setting, incorporates 12 physiological variables, including age and comorbidities, and can yield disproportionately high, distorted scores in older patients.

Imaging-based systems such as the Balthazar grade and the (modified) CT Severity Index depend on contrast-enhanced CT, whose early use is limited because the full extent of pancreatic necrosis often only becomes apparent after the first 48 h, and which carries the additional risks associated with contrast administration [11,12].

A 2022 review by Lee and Cho compared these systems and concluded that no single score reliably predicts severity in the early phase on its own (Ranson AUC 0.810; Glasgow 0.780; APACHE II 0.895; BISAP 0.875), recommending BISAP as the most practical initial tool given its balance of accuracy and simplicity. In a population-based study of 18,256 patients, BISAP performed comparably to APACHE II (AUC 0.82 vs. 0.83) while requiring only five variables available within the first 24 h [13,14]. The Harmless Acute Pancreatitis Score (HAPS) goes further, using only three variables to identify, within about an hour of admission, patients highly likely to have mild disease (specificity 97%, positive predictive value 98%), making it useful for deciding whether a patient can safely remain at a lower-level care facility or requires transfer to a higher level of care [7,15].

Despite this range of options, none of the currently available tools combines admission-only variables, high discriminative accuracy, and the simplicity required for rapid use outside specialized or intensive care settings—a gap that is particularly consequential in primary and regional care centers, where the ability to identify high-risk patients within hours of admission directly determines decisions about monitoring intensity and the need for timely transfer to a tertiary center.

### Aim of the Study

The aim of our study was to develop a new scoring system that is more accurate than the existing scoring systems mentioned above, simple to calculate, applicable already in the emergency setting, and based on parameters available at any local or regional primary care center. We anticipate that this scoring system could be widely applicable and could assist in therapeutic decision-making as well as in determining the appropriate level of patient care, including admission to the intensive care unit, continuous monitoring, or transfer from primary centers to tertiary care facilities.

## 2. Materials and Methods

### 2.1. Study Design and Patients

Our study is a retrospective observational study that uses the clinical, laboratory, and radiological data of patients diagnosed with acute pancreatitis who were admitted to Gastroenterology Department No. 1 of the Târgu Mureș Emergency Clinical Hospital. This was a retrospective chart review; the study protocol, including the predefined study period (1 January–31 December 2025), was approved by the institutional Ethics Committee prior to data extraction (approval number: 18016, approved 23 July 2025), and no study-specific data were accessed before this approval was granted. Data were extracted from electronic medical records only after completion of the full predefined study period, in 2026, once all eligible cases had become available. All patients had provided general written informed consent at the time of hospital admission, in accordance with standard institutional practice, authorizing the use of their anonymized clinical data for research purposes; this general, standing consent—rather than a study-specific prospective or retrospective consent obtained for the present analysis—constituted the ethical basis for the use of patient data in this retrospective study. The study complied with the ethical principles governing research on human subjects, in accordance with the Declaration of Helsinki.

**Figure 1:**
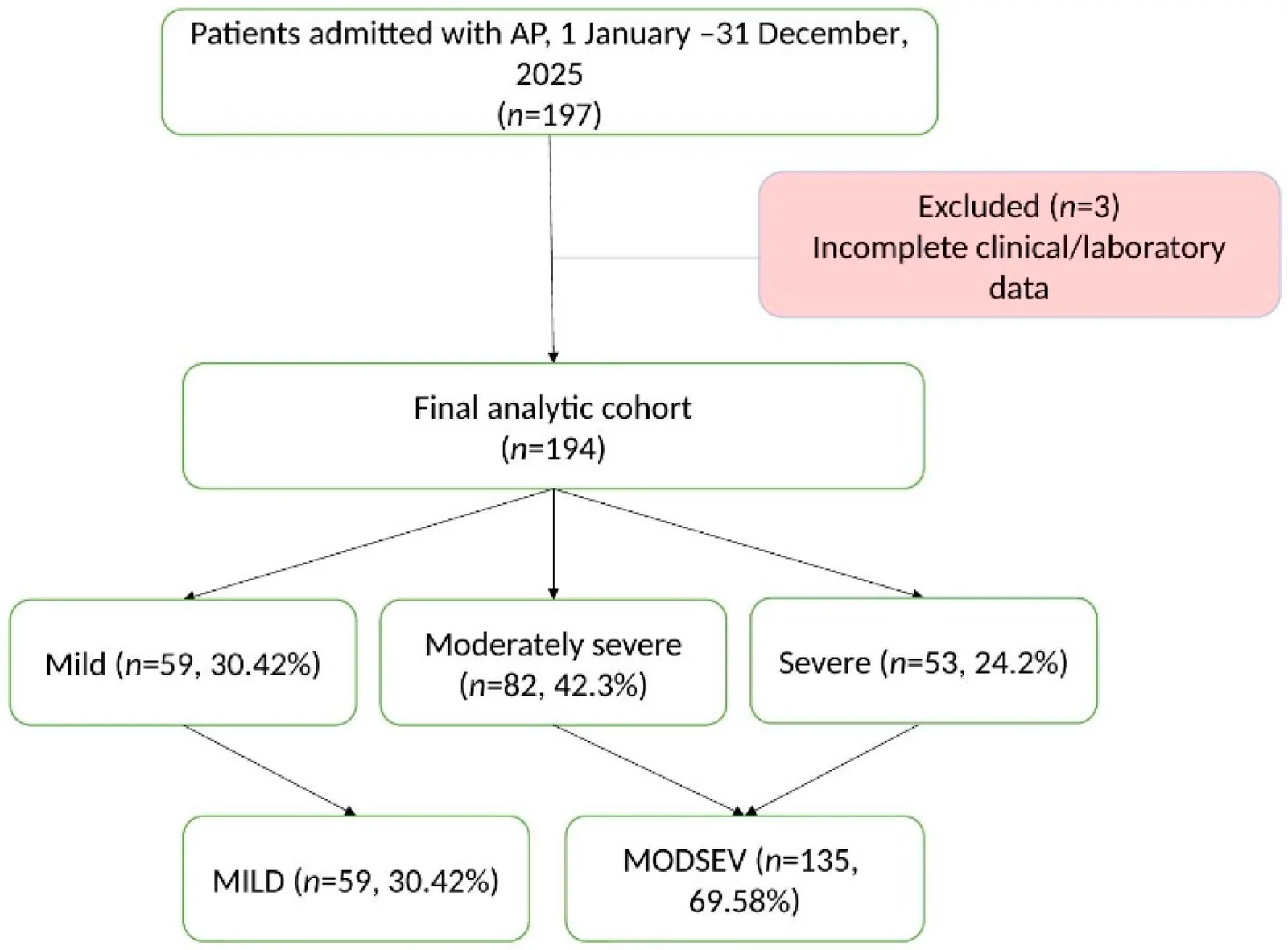
STROBE-style flow diagram of patient screening, exclusion, and final inclusion. Of 197 patients admitted with acute pancreatitis between January 1 and December 31, 2025, 3 were excluded due to incomplete clinical or laboratory data, yielding a final analytic cohort of 194 patients, further stratified by disease severity (mild, moderately severe, severe) according to the Revised Atlanta Classification and collapsed into the binary MILD vs. MODSEV endpoint used for model development.

Patients were recruited consecutively over a one-year period, from 1 January to 31 December 2025. During this period, a total of 197 patients were admitted with a diagnosis of acute pancreatitis; of these, 3 patients (1.5%) were excluded because of incomplete clinical or laboratory data, leaving a final analytic cohort of 194 patients. The patient screening, exclusion, and final inclusion process is summarized in Figure 1 (STROBE-style flow diagram).

No formal a priori sample size or power calculation was performed; all consecutively eligible patients treated during the predefined one-year study period were included, consistent with common practice for single-center retrospective model-development studies, and the resulting events-per-variable ratio is discussed as a limitation below.

Inclusion criteria comprised an age above 18 years and a diagnosis of acute pancreatitis, established according to the criteria of the Revised Atlanta Classification published in 2012. Establishing the diagnosis required at least two of the following three criteria to be met: (1) abdominal pain consistent with acute pancreatitis in the upper abdomen, following exclusion of other possible causes such as perforated gastric or duodenal ulcer, aortic dissection, or myocardial infarction; (2) elevation of serum amylase and/or lipase levels exceeding three times the upper limit of normal; and (3) imaging findings consistent with acute pancreatitis, confirmed by ultrasonography, computed tomography (CT), or magnetic resonance imaging (MRI). In our study, all patients underwent abdominal CT at admission, followed by a follow-up CT scan 24–72 h later. In addition, a chest radiograph was obtained at admission, during which the presence of pleural effusion was recorded as part of data collection. Abdominal ultrasonography was also performed during the hospital stay, allowing the presence of hepatic steatosis to be determined and documented. The diagnosis of fatty liver was established based on the difference in echogenicity between the liver parenchyma and the right renal cortex, with increased hepatic echogenicity considered the ultrasonographic sign of steatosis. We also calculated the FIB-4 score, a four-parameter scoring system used to estimate the risk of liver fibrosis. Based on the resulting score, patients were classified into 3 groups (<1.3 low fibrosis risk, 1.3–2.67 intermediate risk, and >2.67 high fibrosis risk).

### 2.2. Severity Classification

Disease severity was classified according to the Revised Atlanta Classification, which distinguishes three categories: mild, moderately severe, and severe acute pancreatitis. In the mild form, neither organ failure nor local or systemic complications were present. The criterion for the moderately severe form was the presence of transient organ failure lasting less than 48 h and/or the presence of local or systemic complications. Severe acute pancreatitis was defined as organ failure persisting for more than 48 h and involving one or more organ systems. Local complications were defined as the presence of pancreatic and peripancreatic necrosis (sterile or infected), pseudocyst formation, walled-off necrosis (sterile or infected), gastric outlet obstruction, splenic or portal vein thrombosis, and colonic wall necrosis. The development of secondary diabetes mellitus was recorded as a late complication. Systemic complications included the exacerbation of pre-existing comorbidities triggered by acute pancreatitis, such as exacerbation of coronary artery disease or chronic pulmonary disease. The Revised Atlanta Classification is currently the internationally accepted standard for defining and uniformly reporting the severity of acute pancreatitis, and was therefore used in our study to classify disease severity [16].

### 2.3. Etiology Classification

The etiology of acute pancreatitis was determined and classified based on a detailed review of clinical documentation, laboratory and imaging findings, and patient history. Patients were classified into four main etiological groups: (1) biliary acute pancreatitis, in which the pancreatic inflammation was confirmed to be caused by choledocholithiasis or gallstone-induced papillary obstruction; (2) alcohol-induced pancreatitis, confirmed by a history of significant and/or chronic alcohol consumption; (3) metabolic cases, in which hypertriglyceridemia was identified as the underlying cause; and (4) an “other etiology” group, comprising cases that could not be classified into the previous categories, including non-alcoholic toxic (drug-induced), infectious, autoimmune, genetic, idiopathic, post-procedural (e.g., post-ERCP), and structural causes (e.g., pancreas divisum, tumor-related obstruction). When more than one potential etiological factor was identified in a patient, a predefined hierarchical classification algorithm was applied to determine the final etiology: when biliary and alcoholic etiology coexisted, biliary origin was assigned priority; when alcoholic and metabolic (hypertriglyceridemic) etiology coexisted, the metabolic cause was considered dominant; when biliary and metabolic etiology coexisted, biliary origin was again assigned priority. The purpose of applying this hierarchy was to convert the etiological categories into mutually exclusive, analytically well-defined groups, ensuring statistical comparability.

### 2.4. Clinical and Laboratory Data Collection

Several scoring systems are known for assessing the severity of acute pancreatitis (AP). In our study, SIRS, the Ranson score, the Glasgow-Imrie score, the BISAP score, the Balthazar score, the CTSI (Computed Tomography Severity Index), and the HAPS were considered. We calculated only those scoring systems for which the necessary data were available for all enrolled patients. The APACHE II score was not considered, as it could not be calculated for every patient, since some of the required data were unavailable outside the intensive care unit.

Each comparator score was calculated according to its original published definition and corresponding time window. The Ranson and Glasgow-Imrie scores were computed using their respective admission criteria combined with the worst values recorded within the first 48 h of hospitalization, per their standard protocols. The Balthazar grade and CT Severity Index (CTSI) were calculated from contrast-enhanced CT scans, which were performed between 24 and 72 h after admission, in line with current guideline recommendations for optimal visualization of pancreatic necrosis. The BISAP score was calculated using data from the first 24 h of admission, and the HAPS, like ProbScore2, was calculated using data available at the time of admission. Because several of these comparator scores incorporate clinical, laboratory, or imaging information that only becomes available 24 to 72 h after admission, whereas ProbScore2 and HAPS are based exclusively on admission data, the comparison of discriminative performance should be interpreted with this difference in information availability in mind.

Detailed clinical and laboratory data were collected for every patient. Demographic data (sex, age), length of hospital stay, etiology of acute pancreatitis, as well as smoking and alcohol consumption habits were recorded.

Comorbidities included chronic liver disease, heart failure, atrial fibrillation, chronic ischemic heart disease, valvulopathies, acute myocardial infarction, arterial hypertension, chronic kidney disease, bronchial asthma, chronic obstructive pulmonary disease (COPD), osteoarticular diseases, deep vein thrombosis, diabetes mellitus, stroke, and paraneoplastic conditions.

We also recorded proton pump inhibitor (PPI) therapy during hospitalization, the FIB-4 score, the results of gastroscopy performed during the hospital stay, the development of acute respiratory distress syndrome (ARDS), the presence of systemic inflammatory response syndrome (SIRS), and the occurrence of early and late complications.

At admission, we documented vital signs, including body temperature, pulse, blood pressure, and resting respiratory rate, and recorded the clinical presence of peritonitis or acute abdomen. The CONUT score was used to assess nutritional status, and based on the resulting score, patients were classified into 4 groups (0–1 normal nutritional status, 2–4 mild malnutrition, 5–8 points moderate malnutrition, 9–12 severe malnutrition).

The presence of pleural effusion was assessed radiologically using a chest X-ray obtained at the time of hospital admission for every patient. All admission radiographs were interpreted by the hospital’s Radiology Department as part of routine clinical care. Because interpretation followed standard clinical radiological reporting rather than a dedicated, blinded, or duplicate-read research protocol, the timing of image acquisition was consistent across the cohort (at admission), but the formal inter-observer reliability of pleural effusion detection was not separately assessed.

Laboratory investigations included the following parameters: C-reactive protein (CRP) at admission, serum procalcitonin, serum amylase and/or lipase at admission, blood glucose level at admission, and the maximum blood glucose level measured during hospitalization. In addition, serum albumin at admission, the neutrophil-to-lymphocyte ratio (NLR) at admission, 24, and 48 h, and the platelet-to-lymphocyte ratio (PLR) at admission, 24, and 48 h were determined. Additional laboratory parameters included serum cholesterol, lymphocyte count, AST (GOT), ALT (GPT), platelet count, leukocyte count, urea and blood urea nitrogen (BUN), serum creatinine, hematocrit (at admission and at 24 h), as well as serum LDH and calcium. Blood gas analysis was used to determine pO2, base deficit, and the degree of fluid loss.

### 2.5. Statistical Analysis

In this retrospective observational study, we analyzed the clinical, laboratory, and imaging data of patients diagnosed with acute pancreatitis. The data were extracted from the database of Gastroenterology Department No. 1 and subsequently processed using R software (R Foundation for Statistical Computing, Vienna, Austria).

This study is reported in accordance with the TRIPOD (Transparent Reporting of a multivariable prediction model for Individual Prognosis Or Diagnosis) statement for prediction model development studies [17]. A completed TRIPOD checklist, indicating the manuscript section and page addressing each item—including participant flow, missing data handling, the full model specification, calibration, internal validation, and guidance for practical application—is provided in Supplementary Table S3.

The distribution of each continuous variable was assessed using the Shapiro–Wilk test and visual inspection of skewness. Variables with an approximately symmetric distribution (serum albumin) are reported as mean ± SD and compared using Student’s *t*-test. Variables with a skewed distribution (white blood cell count, BUN, admission and peak glucose, platelet-to-lymphocyte ratio, neutrophil-to-lymphocyte ratio, CRP, and creatinine) are reported as median (interquartile range) and compared using the Mann Whitney U test.

Disease severity was determined according to the Revised Atlanta Classification. A binary endpoint was used for the analysis: mild acute pancreatitis versus moderately severe/severe acute pancreatitis.

Potential predictors included in the study comprised demographic characteristics, biochemical and inflammatory markers (e.g., NLR, PLR, CRP, procalcitonin), nutritional and laboratory indices (serum albumin, CONUT score), imaging and morphological severity indicators (Balthazar score, necrosis score), and clinical scores (Ranson, Glasgow-Imrie, BISAP, HAPS), as well as comorbidities and other clinical variables.

Categorical variables were converted into binary indicators, while continuous variables were analyzed in their original form. Missing data were handled using complete-case analysis.

Multicollinearity among predictors was assessed using Spearman’s rank correlation. Strongly correlated variables (|ρ| > 0.69) were considered redundant and were reduced prior to multivariable modeling.

Associations between predictors and the binary endpoint were assessed using Fisher’s exact test for categorical variables and the Wilcoxon rank-sum test for continuous variables. Variables were subsequently ranked according to their discriminative power based on the resulting *p*-values.

A logistic regression model was constructed using the predictor variables to identify patients with moderately severe/severe acute pancreatitis (MODSEV). Variable selection was performed using the exhaustive best-subset method based on the Akaike Information Criterion (AIC), applying the bestglm algorithm.

The discriminative ability of the model was assessed using ROC curve analysis. AUC (area under the curve) values were calculated using the pROC package.

To assess the extent to which the model’s apparent performance might be optimistic as a result of being developed and evaluated in the same cohort, internal validation was additionally performed using bootstrap resampling (1000 replicates) with optimism correction of the C-statistic (Harrell’s method) and using repeated (100×) 10-fold cross-validation, applying the same six predictors and the same logistic regression modeling approach as in the original model.

Calibration of the model was assessed in addition to discrimination, using the Brier score, calibration-in-the-large, and the calibration slope (obtained from a logistic recalibration model regressing the observed outcome on the linear predictor), together with a graphical calibration plot comparing predicted probabilities with observed outcome frequencies. Optimism-corrected estimates of the Brier score and calibration slope were obtained using the same bootstrap resampling procedure (1000 replicates) applied to the discrimination analysis.

The performance of the model was compared with conventional clinical severity scores, including the Ranson score, Glasgow-Imrie score, Balthazar grade and score, BISAP score, and HAPS.

In this section, the authors declare that no generative artificial intelligence (GenAI) tools were used for study design, data collection, analysis, or interpretation. GenAI-assisted tools were used only for superficial text and formatting editing, which does not require disclosure.

## 3. Results

### 3.1. Baseline Characteristics of the Study Population

**Figure 2:**
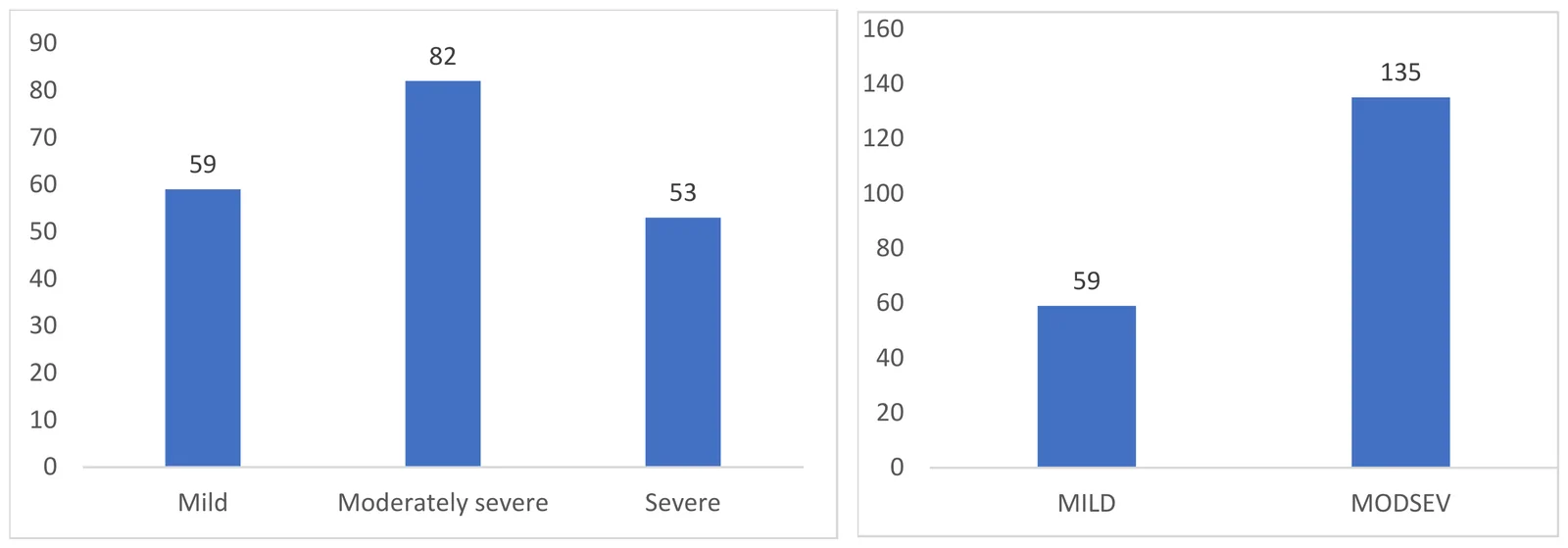
Distribution of acute pancreatitis patients according to disease severity (Revised Atlanta Classification): mild (*n* = 59, 30.4%), moderately severe (*n* = 82, 42.3%), and severe (*n* = 53, 24.2%); respectively the comparison of the mild group with the combined group of moderately severe/severe cases (MODSEV).

A total of 194 patients were enrolled in the study, stratified by disease severity into mild (*n* = 59, 30.42%), moderately severe (*n* = 82, 42.3%), and severe (*n* = 53, 24.2%) forms, based on the Revised Atlanta Classification. During the analysis, binary endpoints were applied; consequently, mild (MILD) cases were compared with the combined group of moderately severe/severe cases (MODSEV) (Figure 2).

The baseline demographic, clinical and anamnestic, hemodynamic, nutritional, and laboratory characteristics of the study population are summarized in Table 1. Additional comorbidity, endoscopy, medication, etiology, and toxic-habit characteristics are summarized in Supplementary Table S1.

Comparing the demographic aspects of the two groups, there were no significant differences in age (54.68 ± 16.32 vs. 57.30 ± 16.64 years; *p* = 0.270) or in gender, with a similar proportion of males (62.7% vs. 65.2%). This is likely due to the small sample size.

The etiological distribution of acute pancreatitis was comparable between the two groups (*p* = 0.849). Biliary etiology was the leading cause in MILD (39.0%) and MODSEV (37.0%) patients, followed by alcohol-induced pancreatitis (25.4% vs. 24.4%), metabolic-associated pancreatitis (20.3% vs. 25.9%), and other causes (15.3% vs. 12.6%). The absence of significant between-group differences in etiology suggests that disease progression in this cohort is not primarily etiology-dependent.

Similarly, the distribution of toxic habits did not differ significantly between groups (*p* = 0.408). Combined alcohol and tobacco use was numerically more prevalent in the MODSEV group (31.9% vs. 20.3%), while isolated alcohol and smoking exposure were approximately the same. Although statistical significance was not achieved, the trend towards higher combined toxic exposure in the MODSEV group warrants further investigation, given the established synergistic role of alcohol and tobacco in pancreatic injury.

Hypertension was the most prevalent cardiovascular comorbidity and was significantly more frequent in the MODSEV group (68.1% vs. 52.5%; *p* = 0.038). A non-significant trend toward a greater burden of chronic ischemic cardiopathy was observed in MODSEV patients (16.3% vs. 6.8%; *p* = 0.073). Other cardiovascular conditions, including atrial fibrillation (7.4% vs. 3.4%; *p* = 0.285), acute heart failure (12.6% vs. 8.5%; *p* = 0.405), valvular disease (9.6% vs. 11.9%; *p* = 0.638), deep vein thrombosis (6.7% vs. 1.7%; *p* = 0.150), and prior acute myocardial infarction (3.7% vs. 1.7%; *p* = 0.457), were numerically more common in the MODSEV group but did not reach statistical significance.

Diabetes mellitus was present in 26.7% of MODSEV patients compared to 15.3% in the MILD group, with a trend toward significance (*p* = 0.083). Similarly, chronic kidney disease (CKD) was more prevalent in the MODSEV group (17.0% vs. 6.8%; *p* = 0.058), approaching statistical significance. These findings are consistent with the fact that metabolic comorbidities increase the risk of more severe disease.

Liver steatosis was highly prevalent in both groups, affecting 81.5% of MODSEV and 69.5% of MILD patients (*p* = 0.064). Fibrosis risk, assessed by the FIB-4 score, did not differ significantly between groups (*p* = 0.501): high-risk fibrosis was identified in 50.8% of MILD and 48.9% of MODSEV patients, indeterminate risk in 18.6% and 25.9%, and low risk in 30.5% and 25.2%, respectively. The high burden of advanced fibrosis across both groups highlights the importance of metabolic liver disease as a comorbidity in this population.

The prevalence of chronic respiratory disease (COPD and asthma) was similar between the two groups (14.8% vs. 13.6%; *p* = 0.819). However, pleural effusion was significantly more frequent in the MODSEV group (43.7% vs. 13.6%; *p* < 0.001), reflecting higher systemic inflammation and fluid redistribution. Systemic inflammatory response syndrome (SIRS) was recorded in a significantly higher proportion of MODSEV patients (64.9% vs. 43.1%; *p* = 0.005). ARDS was numerically more frequent in MODSEV patients (11.9% vs. 3.4%; *p* = 0.062), although without reaching statistical significance, probably due to limited sample size.

Upper gastrointestinal endoscopy was performed in 37.3% of MILD and 26.7% of MODSEV patients (*p* = 0.137) during the hospital stay. Gastritis was the most frequently identified lesion, numerically more common in the MILD group (27.1% vs. 15.6%; *p* = 0.059). Statistically significant differences were observed for lesions described at the level of the cardia (6.8% vs. 0.7%; *p* = 0.015), fundus (6.8% vs. 0%; *p* = 0.002), and duodenal second portion (6.8% vs. 0.7%; *p* = 0.015), all more frequent in the MILD group. Endoscopically identified tumors reached the threshold for statistical significance (5.1% vs. 0.7%; *p* = 0.050). Esophagitis (15.3% vs. 10.4%; *p* = 0.333), erosions, gastropathy, ulcers, and Mallory-Weiss lesions were comparable between groups. Proton pump inhibitor therapy was administered in 74.6% of MILD and 83.0% of MODSEV patients (*p* = 0.176), without an apparent impact on disease severity.

All measured inflammatory markers were significantly elevated in the MODSEV group at admission. White blood cell count was higher in MODSEV patients (15.11 ± 6.26 vs. 11.09 ± 4.11 × 10^3^/µL; *p* < 0.001), as was C-reactive protein (120.57 ± 126.97 vs. 56.58 ± 72.39 mg/L; *p* < 0.001). The neutrophil-to-lymphocyte ratio (NLR) at admission was more than twice as high in the MODSEV group (14.91 ± 13.08 vs. 6.69 ± 3.99; *p* < 0.001), and this elevation persisted at 24 h (12.88 ± 11.73 vs. 5.28 ± 3.60; *p* < 0.001) and 48 h (10.75 ± 9.66 vs. 4.06 ± 2.20; *p* < 0.001), indicating more pronounced and persistent systemic inflammation. Platelet-to-lymphocyte ratio (PLR) was similarly elevated at all three time points in the MODSEV group (all *p* ≤ 0.001).

Hematocrit levels at admission and at 24 h did not differ significantly between the groups (*p* = 0.233 and *p* = 0.624, respectively). Platelet counts were also comparable (228.33 ± 89.86 vs. 222.77 ± 89.99 × 10^3^/µL; *p* = 0.785). In contrast, lymphocyte counts at admission were significantly lower in the MODSEV group (1286 ± 997.63 vs. 1427.95 ± 611.92 × 10^3^/µL; *p* = 0.008), consistent with the elevated NLR observed in this group.

Nutritional impairment was significantly more prevalent in the MODSEV group. Serum albumin at admission was lower in MODSEV patients (3.46 ± 0.55 vs. 3.88 ± 0.37 g/dL; *p* < 0.001), as was the total CONUT score (4.32 ± 2.90 vs. 2.73 ± 2.02; *p* = 0.001). Categorical analysis of the CONUT score revealed a significantly worse nutritional profile in MODSEV patients (*p* = 0.001): normal nutritional status was present in only 14.8% of MODSEV patients versus 30.5% of MILD patients, while moderate-to-severe malnutrition was identified in 40.0% of MODSEV patients compared to 13.6% in the MILD group. Total cholesterol levels did not differ significantly between groups (169.49 ± 68.51 vs. 176.32 ± 76.32 mg/dL; *p* = 0.748).

Blood urea nitrogen (BUN) at admission was significantly elevated in the MODSEV group (23.68 ± 18.05 vs. 16.10 ± 8.72 mg/dL; *p* = 0.001), indicating greater renal stress in more severe cases. Serum creatinine was paradoxically higher in the MILD group (1.88 ± 7.84 vs. 1.15 ± 0.73 mg/dL; *p* = 0.004), likely attributable to outlier values reflected in the high standard deviation of that group. Hepatic transaminases such as AST (177.64 ± 219.53 vs. 168.38 ± 239.16 U/L; *p* = 0.411) and ALT (153.68 ± 204.09 vs. 137.34 ± 197.76 U/L; *p* = 0.454) did not differ significantly between groups.

Serum amylase at admission was comparably elevated in both groups (MILD: 1048.12 ± 1501.69 U/L; MODSEV: 1019.69 ± 1135.67 U/L; *p* = 0.404), confirming uniform elevation as a diagnostic feature irrespective of disease severity. In contrast, glycemic parameters were significantly worse in the MODSEV group: both admission blood glucose (170.39 ± 118.33 vs. 131.71 ± 57.35 mg/dL; *p* = 0.003) and peak glucose during hospitalization (187.48 ± 140.26 vs. 134.55 ± 58.32 mg/dL; *p* < 0.001) were substantially higher, indicating more pronounced metabolic dysregulation in severe cases.

Vital signs at admission, including body temperature, heart rate, respiratory rate, and systolic and diastolic blood pressure, did not differ significantly between the groups (*p* > 0.05). Blood pressure category distribution was also similar (*p* = 0.740), with Grade II hypertension being the most common category in both groups (28.8% MILD vs. 31.1% MODSEV).

Disease severity was strongly associated with adverse clinical outcomes. Patients in the MODSEV group were hospitalized significantly longer than those in the mild group (9.61 ± 5.25 vs. 6.97 ± 1.99 days; *p* < 0.001), indicating increased healthcare costs and higher severity. In-hospital mortality occurred exclusively among MODSEV patients, with 14 deaths (11.57%), whereas no deaths were recorded in the mild group (*p* = 0.01).

Complications also differed markedly between groups. Uncomplicated cases were more frequent in the mild group (20/59, 33.9%) compared with the MODSEV group (26/135, 19.3%). Peripancreatic fluid collections were the most common complication in both groups but were substantially more frequent in mild patients numerically (33/59, 55.9% vs. 49/135, 36.3%). Necrotic collections showed the most pronounced disparity, occurring predominantly in the MODSEV group (51/135 cases, 37.77%) and being rare in mild disease (3/54, 5.6%), highlighting a strong association with the moderately severe/severe (MODSEV) outcome. Pseudocysts were observed only in the MODSEV group (6/135, 4.4%), while de novo diabetes occurred at a similarly low frequency in both groups (3/59, 5.1% in mild vs. 3/135, 2.2% in MODSEV). Overall, complication patterns demonstrated a clear shift toward more structurally severe pancreatic sequelae in the MODSEV group, particularly necrotic collections, reflecting greater disease severity and systemic involvement.

Detailed comorbidity, endoscopic, medication, etiology, and toxic-habit data for the study population—including cardiovascular, respiratory, metabolic, renal, hepatic, and neurological comorbidities, and upper gastrointestinal endoscopy findings—are provided in the Supplementary Materials (Table S1). Of these, pleural effusion (43.7% vs. 13.6%, *p* < 0.001) and SIRS (64.9% vs. 43.1%, *p* = 0.005) are of direct relevance to ProbScore2 and are discussed further below; hypertension (68.1% vs. 52.5%, *p* = 0.038) was also significantly more frequent in the MODSEV group.

These findings demonstrate that MODSEV patients present with a significantly more severe clinical, inflammatory, nutritional, and metabolic profile than MILD patients. The most robust discriminators between severity groups at admission were inflammatory markers (NLR, PLR, WBC, and CRP), nutritional indices (albumin, CONUT score), glycemic status, renal stress indices (BUN, serum creatinine), complication burden (SIRS, pleural effusion), lymphocyte count, hospitalization length, and the presence of hypertension in the medical history (Figure S1).

Boxplots comparing the MILD and MODSEV groups across the significant admission discriminators identified in this analysis are provided in the Supplementary Materials (Figure S1).

### 3.2. Comparison with Established Severity Scoring Systems

**Figure 3:**
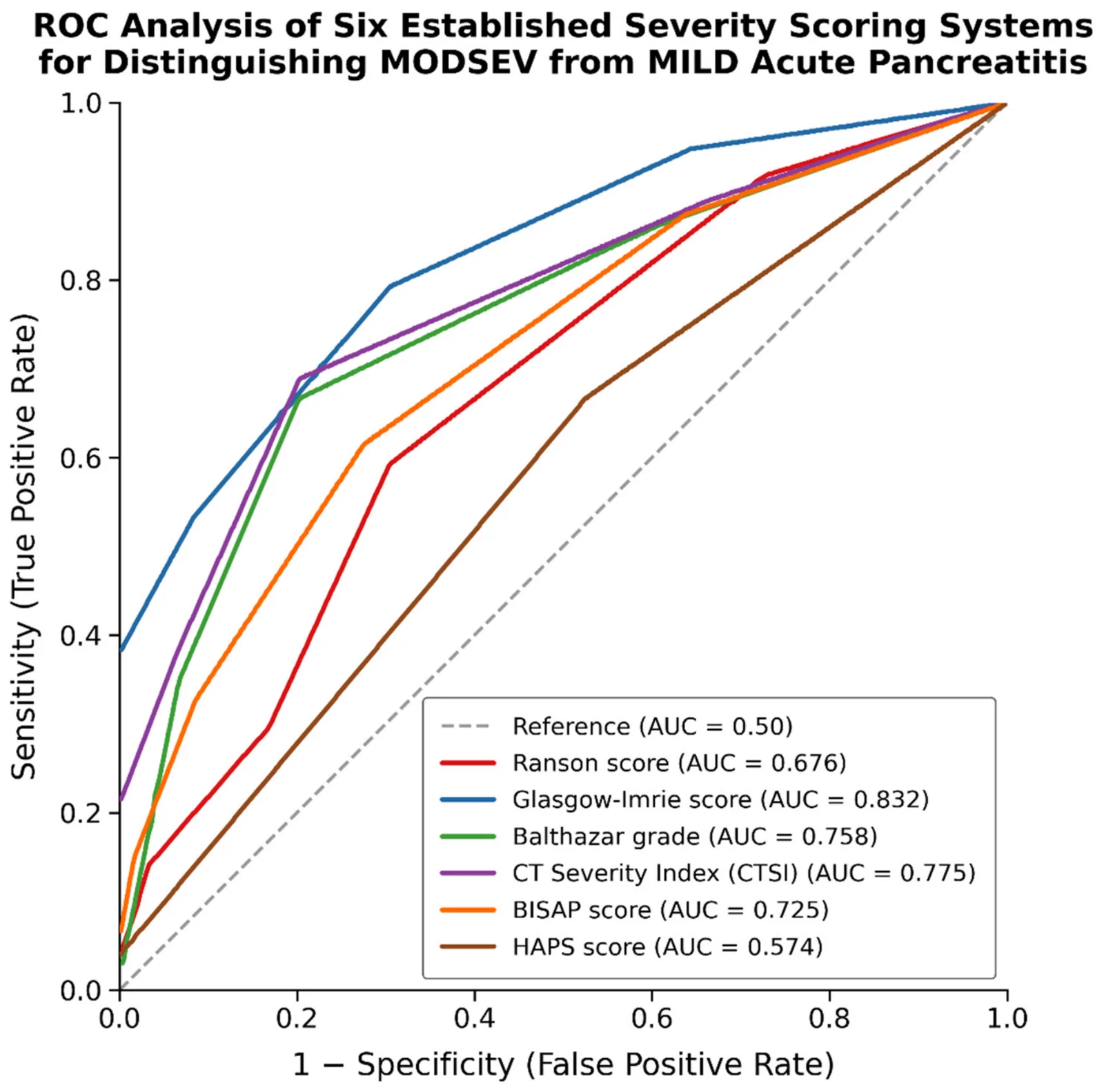
ROC analysis of six established severity scoring systems for distinguishing MODSEV from MILD acute pancreatitis.

We evaluated six established severity scoring systems (Ranson, Glasgow-Imrie, Balthazar, CTSI, BISAP, and HAPS) for predicting moderately severe/severe acute pancreatitis. Diagnostic accuracy was compared by calculating the area under the receiver operating characteristic curve (AUC) for each system (Figure 3).

The Glasgow-Imrie score demonstrated the highest discriminative performance among all evaluated conventional scoring systems, with an AUC of 0.832 (95% CI: 0.765–0.883, *p* < 0.001), indicating good diagnostic accuracy; at the optimal Youden Index threshold, its sensitivity was 74.6% and specificity was 74.4%. The CTSI score achieved the second highest AUC of 0.775 (95% CI: 0.707–0.842, *p* < 0.001), with an optimal sensitivity of 70.2% and specificity of 70.4%. The Balthazar score followed closely, with an AUC of 0.758 (95% CI: 0.685–0.829, *p* < 0.001) and a sensitivity/specificity of 68.9%/68.8% at the optimal cut-off; these two radiological scores are complementary and are commonly used together in clinical practice.

The BISAP score showed moderate discriminative accuracy (AUC = 0.725, 95% CI: 0.640–0.792, *p* < 0.001; sensitivity 65.5%, specificity 65.8%), followed by the Ranson score (AUC = 0.676, 95% CI: 0.584–0.752, *p* < 0.001; sensitivity 61.8%, specificity 62.3%).

Among the evaluated scoring systems, the HAPS demonstrated the lowest discriminative performance, with an AUC of 0.574 (95% CI: 0.497–0.672), which did not reach statistical significance (*p* = 0.064), suggesting limited utility for predicting disease severity in the present cohort. This may be explained by the absence of peritonitis in the study population, a key component of this scoring system.

### 3.3. Development of the New Prognostic Score (ProbScore2)

To develop the new prognostic score, 12 parameters with robust discriminatory value were selected from the previously identified candidate variables. The inclusion criteria were determined subjectively, with the primary consideration being the routine availability of these parameters at hospital admission. The selected variables were: C-reactive protein (CRP), white blood cell count (WBC), presence of systemic inflammatory response syndrome (SIRS), blood urea nitrogen (BUN), history of hypertension (HTN), admission blood glucose level, presence of pleural effusion, neutrophil-to-lymphocyte ratio (NLR), lymphocyte count, serum albumin, platelet-to-lymphocyte ratio (PLR), and serum creatinine. This pre-selection reflected practical clinical availability at admission (prioritizing routine bloodwork, vital signs, and a chest radiograph over specialized or delayed investigations) combined with variables previously reported as significant discriminators of AP severity in the univariable analysis described above (Section 3.2) or in the existing literature, rather than an unrestricted, purely data-driven search across all measured parameters. This strategy was intended to reduce the risk of chance findings from testing a very large number of candidate variables in a moderate-sized single-center cohort, at the cost of an element of subjectivity in the initial candidate list; we address this trade-off further below with a penalized-regression sensitivity analysis.

Missing data among the 12 candidate variables were minimal: platelet-to-lymphocyte ratio was missing in 3 patients (1.5%), SIRS status in 2 patients (1.0%), and CRP in 1 patient (0.5%). All other candidate variables (white blood cell count, BUN, hypertension, admission glucose, pleural effusion, NLR, lymphocyte count, albumin, and creatinine) were complete for all 194 patients. Overall, 6 of 194 patients (3.1%) had at least one missing value among the 12 candidates and were excluded via listwise deletion from the initial backward stepwise selection model (*n* = 188). The final six-variable model was subsequently evaluated in all patients with complete data for the six retained predictors (*n* = 191).

Among the 12 candidate variables, independent predictors were identified using binary logistic regression with a backward stepwise, likelihood-ratio elimination procedure (entry criterion: *p* < 0.05; removal criterion: *p* > 0.10; classification cut-off = 0.5). The initial (full) model included all 12 variables, with disease severity (mild vs. moderately severe/severe) as the dependent variable.

After seven elimination steps, the following variables were sequentially removed based on their minimal contribution to the model: NLR (step 2), SIRS (step 3), lymphocyte count (step 4), creatinine (step 5), hypertension (step 6), and CRP (step 7). Model performance remained stable throughout the elimination process (Nagelkerke R^2^ decreased only from 0.514 to 0.496), indicating that removal of these variables did not substantially reduce the explanatory power of the model.

Following backward elimination, six variables remained in the final model: white blood cell count, BUN, pleural effusion, admission glucose, platelet-to-lymphocyte ratio at admission, and serum albumin. These variables were subsequently re-evaluated using a confirmatory logistic regression model with the ENTER method, yielding the final coefficients shown in Table 2.

As a sensitivity analysis addressing the potential instability of stepwise variable selection in a moderate-sized cohort, we additionally fitted an L1-penalized (LASSO) logistic regression model across the same 12 candidate variables, with the penalty strength selected via 10-fold cross-validation using the one-standard-error rule. This penalized model retained six variables (white blood cell count, CRP, hypertension, pleural effusion, neutrophil-to-lymphocyte ratio, and albumin; in-sample AUC = 0.850); three of these (white blood cell count, pleural effusion, and albumin) were identical to those retained by backward stepwise selection, while the remaining three differed (LASSO retained CRP, hypertension, and NLR, whereas backward stepwise retained BUN, admission glucose, and PLR), most likely reflecting collinearity among inflammatory and metabolic markers measured at admission. Despite this partial divergence in the selected variables, both approaches achieved similar discriminative performance, and the bootstrap-based internal validation reported in Section 2.5 already provides an optimism-corrected estimate of the final model’s performance that does not depend on which specific variable-selection method was used.

The final model demonstrated good calibration (Hosmer–Lemeshow test: χ^2^ = 4.87, df = 8, *p* = 0.771), with a Nagelkerke R^2^ of 0.478 and an overall classification accuracy of 82.7%. Because the Hosmer–Lemeshow test alone is an insufficient basis for assessing calibration, a more comprehensive evaluation—including a calibration plot, calibration slope and intercept, Brier score, and decision curve analysis—is reported later in the Results (Section 3.5).

To allow the model to be applied directly to individual patients, the full fitted logistic regression equation is provided here. The predicted logit (log-odds) of moderately severe/severe acute pancreatitis (MODSEV) is logit(*p*) = 3.083 + 0.1776 × (white blood cell count, in 1000 cells/µL) + 0.0469 × (BUN, mg/dL) + 0.947 × (pleural effusion: 1 = present, 0 = absent) + 0.0098 × (admission glucose, mg/dL) + 0.0037 × (PLR at admission) − 2.068 × (serum albumin, g/dL). The predicted probability is then obtained as *p* = 1/(1 + e − logit(*p*)). This full formula, together with the simplified 0–6 point bedside score described below, allows the model to be applied at the individual patient level using either electronic calculation (full formula) or a manual bedside count (simplified score).

Serum albumin (lower values indicating greater disease severity; OR = 0.12), glucose level at admission, and blood urea nitrogen emerged as the strongest statistically significant predictors (*p* < 0.05). Pleural effusion (*p* = 0.058) and platelet-to-lymphocyte ratio at admission (*p* = 0.070) did not reach the conventional *p* < 0.05 threshold for statistical significance in the final model and should not be interpreted as independent statistically significant predictors on their own. They were retained because they satisfied the pre-specified removal criterion of the backward stepwise procedure (*p* > 0.10), which was defined before model fitting, and because of their established clinical relevance to acute pancreatitis severity. To assess whether their retention was justified beyond this pre-specified statistical rule, we compared the full six-variable model with a reduced four-variable model excluding both pleural effusion and PLR: the two variables jointly contributed significantly to model fit (likelihood ratio test, χ^2^ = 8.98, df = 2, *p* = 0.011) and their inclusion improved both discrimination (AUC 0.873 vs. 0.852) and model fit (AIC 168.5 vs. 173.5) relative to the reduced model, indicating that although neither variable was independently significant, their joint contribution to the model was not attributable to chance.

The discriminative performance of the new six-variable score (ProbScore2) was evaluated using ROC analysis (Table 3). ProbScore2 alone demonstrated excellent discriminative ability for predicting moderately severe/severe acute pancreatitis.

An area under the ROC curve of 0.873 indicates excellent discriminatory performance, according to the widely accepted classification in which an AUC > 0.80 represents excellent diagnostic accuracy.

To quantify the degree of overfitting expected from same-cohort development and evaluation, internal validation of ProbScore2 was performed using bootstrap resampling. Across 1000 bootstrap replicates, the mean optimism in the AUC was 0.020, yielding an optimism-corrected AUC of 0.856. This estimate closely agreed with the AUC obtained from repeated (100×) 10-fold cross-validation (mean AUC = 0.853, SD = 0.009; 2.5th–97.5th percentile range: 0.836–0.870). The small difference between the apparent (0.873) and internally validated (0.853–0.856) AUC values indicates only modest overfitting and supports the internal robustness of ProbScore2, although these internal estimates cannot substitute for external validation in an independent cohort.

For each continuous variable included in the final model (white blood cell count, BUN, admission glucose, PLR, and serum albumin), optimal, clinically applicable cut-off values were determined using ROC analysis by maximizing the Youden Index (sensitivity − [1 − specificity]) (Table 4).

**Figure 4:**
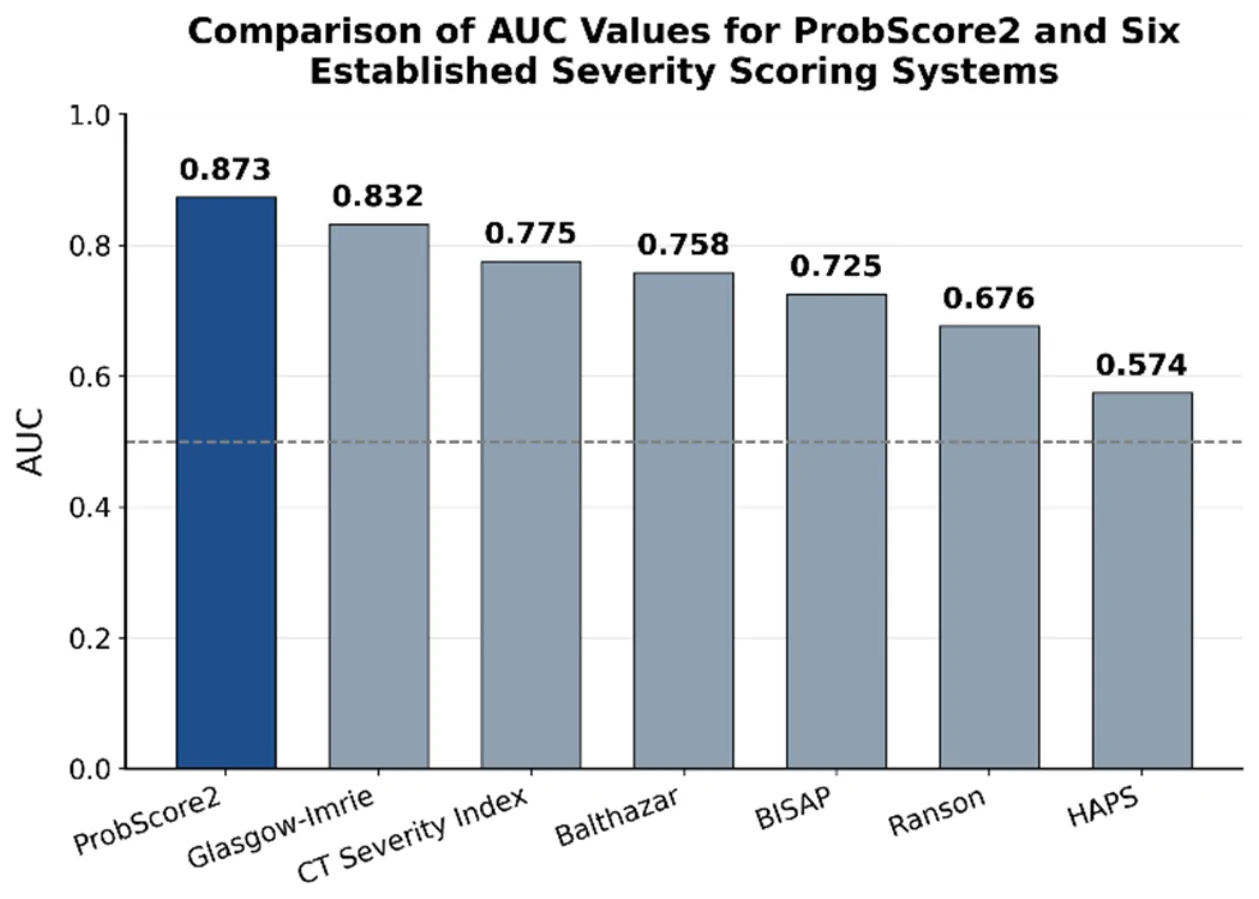
Comparison of AUC values for ProbScore2 and six established severity scoring systems calculated in our cohort.

The discriminatory performance of the new score was directly compared with the established severity scoring systems described above (Table 5, Figure 4).

Within this cohort, ProbScore2 achieved a numerically higher AUC (0.873) than the Glasgow-Imrie score (0.832), CTSI (0.775), Balthazar score (0.758), BISAP (0.725), Ranson (0.676), and HAPS (0.574); these comparisons were made within the same retrospective, single-center sample used for model development and were not adjusted for multiple testing. This numerical difference was particularly pronounced when comparing ProbScore2 to the traditionally less discriminative clinical scoring systems, including HAPS, Ranson, and BISAP, within this cohort.

### 3.4. Performance of the Simplified Bedside Score (0–6 Points)

Based on the threshold values shown in Table 4, each of the six variables can be assigned a binary score (0 or 1), allowing the construction of a simple, unweighted bedside clinical scoring system alongside the original ProbScore2 logistic regression formula. One point was assigned for each of the six dichotomized criteria (total score range 0–6) for all patients in the cohort (*n* = 194), and diagnostic accuracy was evaluated using ROC analysis (Table 6).

The results demonstrated a clear, stepwise association between the score and the probability of a moderately severe/severe (MODSEV) outcome: 15.4% of patients with a score of 0 were classified as moderately severe/severe (MODSEV), whereas this proportion increased to 100% among those with a score ≥5.

**Figure 5:**
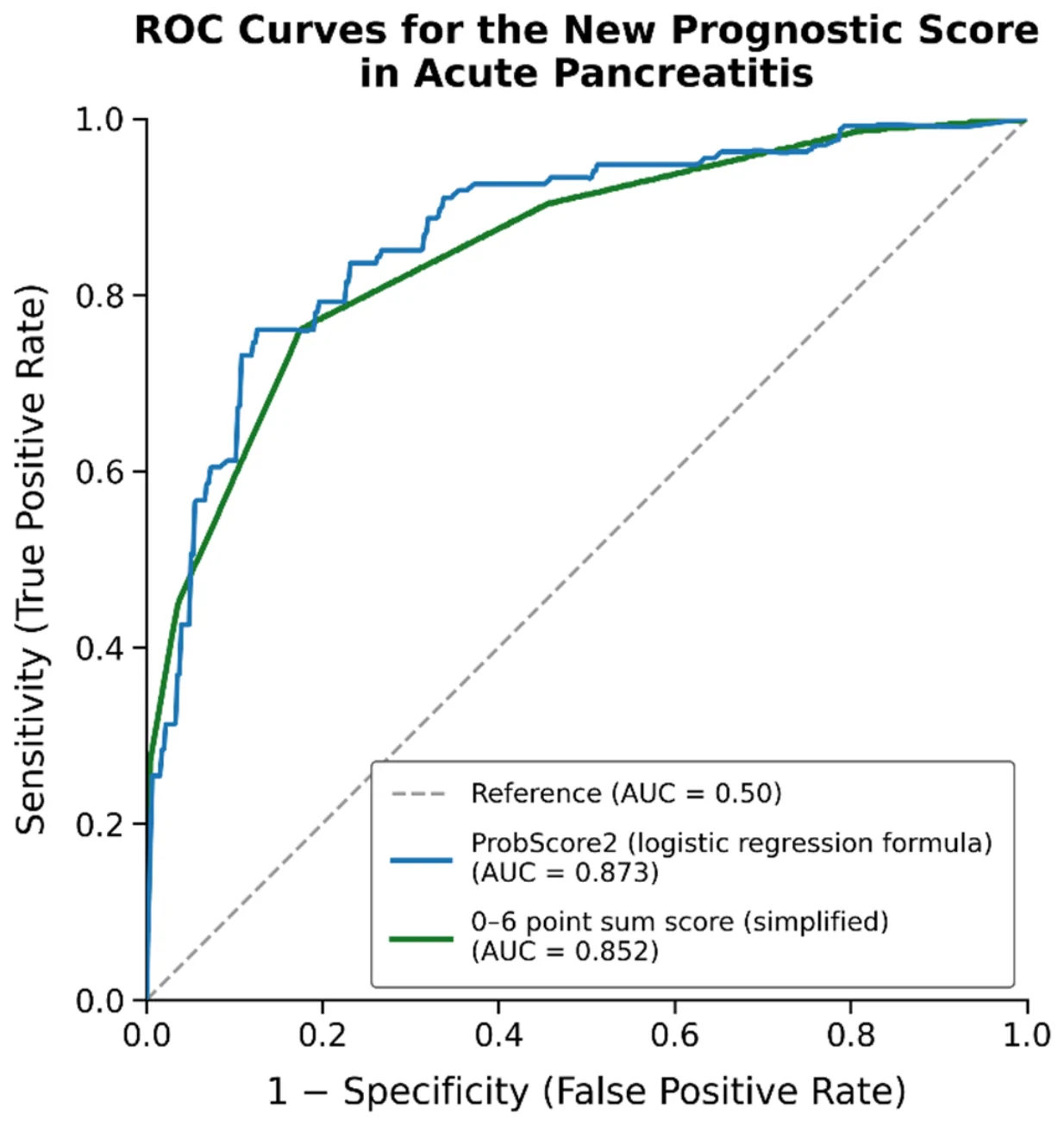
ROC curves for the ProbScore2 logistic regression model and the simplified 0–6 point bedside score.

The simplified bedside score demonstrated excellent discriminatory ability, with an AUC of 0.852 (95% CI: 0.797–0.902, bootstrap estimate). The optimal threshold, based on the maximum Youden Index (0.567), was ≥3 points, corresponding to a sensitivity of 77.0% and a specificity of 79.7% (Table 7, Figure 5).

The 95% CI reported above reflects sampling uncertainty around the apparent AUC but does not account for the fact that the six cut-off values (Table 4) were themselves derived by maximizing the Youden Index in the same cohort subsequently used to evaluate the simplified score. This circularity can inflate apparent performance. To address this explicitly, we repeated the full cut-off derivation and scoring procedure within a bootstrap resampling framework (1000 replicates): in each replicate, Youden-optimal cut-offs were re-derived from the bootstrap sample, the simplified score was recomputed accordingly, and its AUC was evaluated both in the bootstrap sample and in the original cohort. This yielded a mean optimism of 0.026 and an optimism-corrected AUC of 0.827 for the simplified bedside score, compared with the apparent AUC of 0.853—a larger correction than that observed for the full logistic regression model (apparent 0.873 vs. internally validated 0.853–0.856), consistent with the additional optimism introduced by data-driven cut-off selection. The individual cut-offs varied in their bootstrap stability: those for white blood cell count, admission glucose, PLR, and albumin were relatively stable (bootstrap median equal to or close to the apparent cut-off), whereas the BUN cut-off was less stable (apparent 15.0 mg/dL; bootstrap median 18.6 mg/dL; 95% range 15.0–23.0 mg/dL), indicating that this particular threshold should be interpreted with caution until confirmed in an independent cohort.

When evaluated in the same cohort of 194 patients, the simplified score achieved an AUC of 0.852, only marginally lower than that of the full logistic regression model (ProbScore2; AUC = 0.873), indicating that the unweighted additive score preserves most of the predictive performance of the original model.

**Figure 6:**
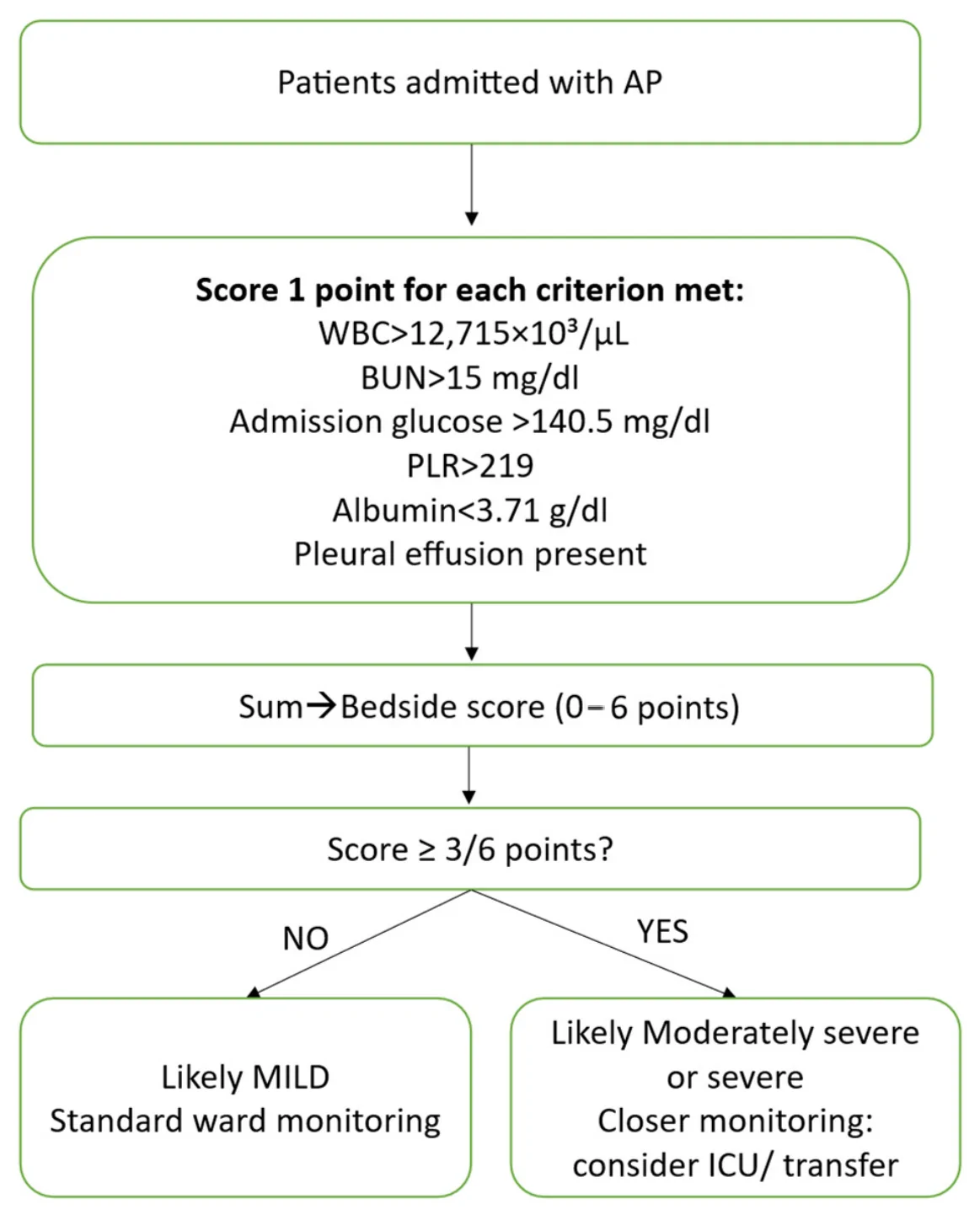
Schematic overview of the simplified ProbScore2 bedside score as applied in routine clinical practice.

Overall, fulfillment of at least three of the six criteria (≥3/6)—white blood cell count >12.72 ×10^3^/µL, BUN >15.0 mg/dL, admission glucose >140.5 mg/dL, PLR >219, serum albumin <3.71 g/dL, and/or presence of pleural effusion—represents the optimal statistical threshold for identifying patients at risk of developing moderately severe/severe acute pancreatitis. At this cut-off, approximately 77% of patients with moderately severe/severe acute pancreatitis (MODSEV) are correctly identified, while approximately 80% of patients with mild disease are correctly excluded. Higher scores (≥4–5) provide greater specificity (96.6–100%) and may therefore be useful for very high-risk patients, although their reduced sensitivity makes them less suitable as primary decision thresholds (Figure 6).

### 3.5. Calibration and Decision Curve Analysis

**Figure 7:**
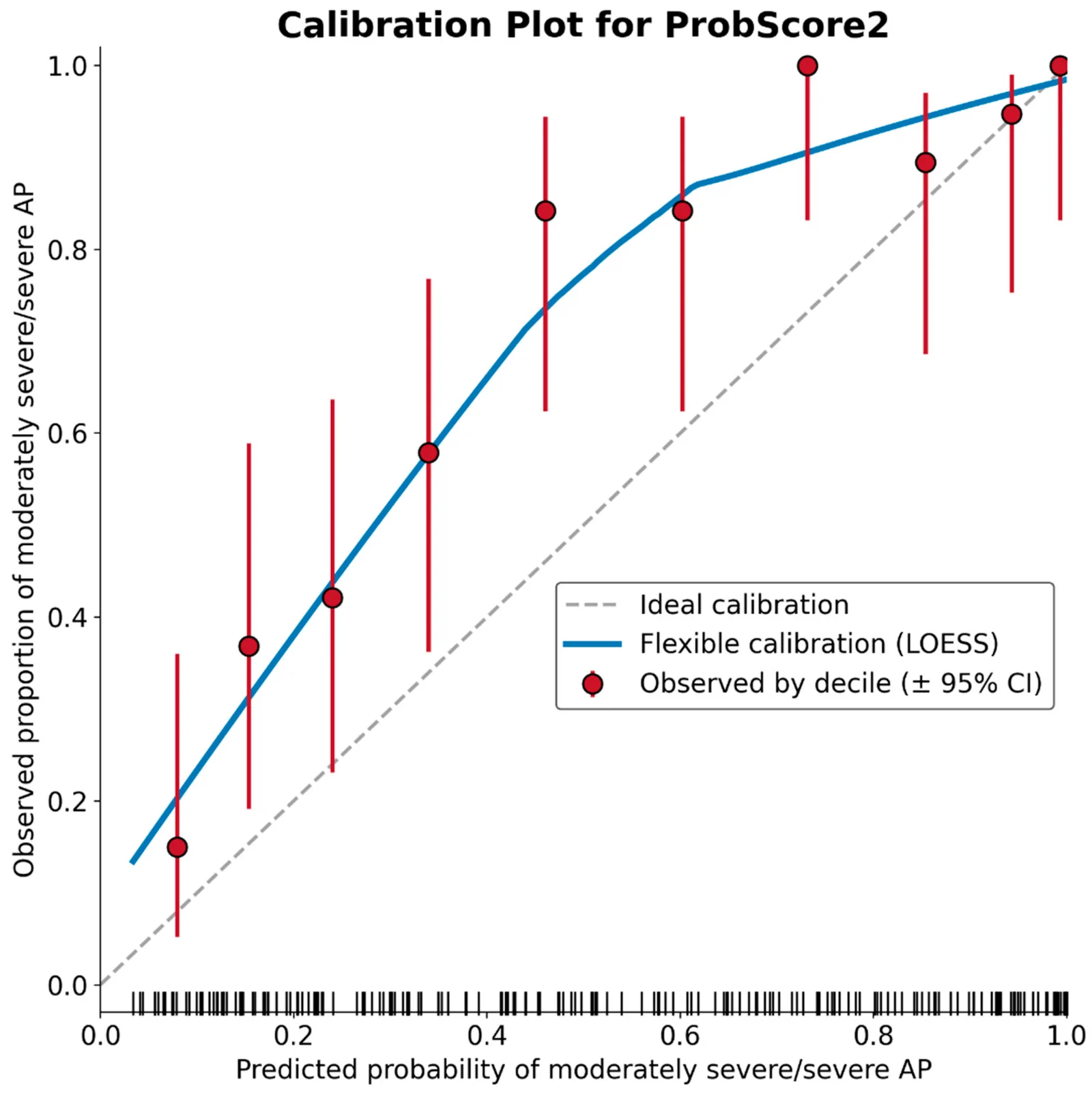
Calibration plot of ProbScore2. The dashed diagonal line represents perfect calibration. The solid blue line shows the flexible (LOESS) calibration curve. Red points show observed proportions (±95% CI) within deciles of predicted risk. Tick marks along the x-axis show the distribution of predicted probabilities.

In addition to discrimination, the calibration of ProbScore2 was evaluated. The apparent Brier score was 0.128 (null-model/base-rate Brier score = 0.209; scaled Brier score = 0.389), and by construction the apparent calibration-in-the-large was 0 and the apparent calibration slope was 1.0 when evaluated in the same data used to fit the model. After bootstrap optimism correction (1000 replicates), the Brier score was 0.141 (scaled Brier score = 0.328) and the calibration slope was 0.867, indicating that a shrinkage factor of approximately 0.87 would be advisable if the model coefficients were to be applied prospectively, consistent with the modest optimism already noted for discrimination. The agreement between predicted probabilities and observed outcome frequencies is shown graphically in Figure 7, based on a locally weighted (LOESS) calibration curve together with observed proportions and 95% confidence intervals across deciles of predicted risk.

**Figure 8:**
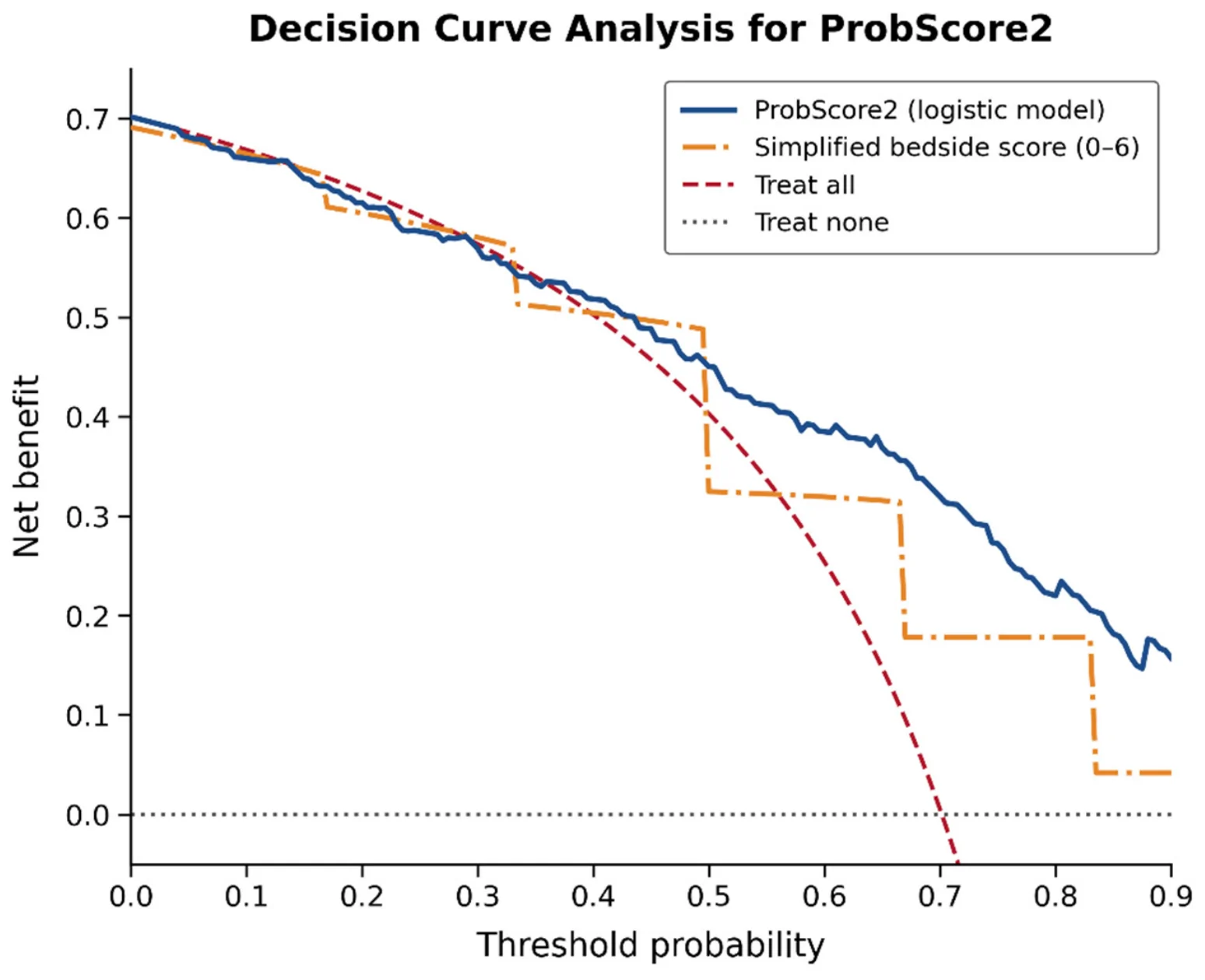
Decision curve analysis for ProbScore2. Net benefit is plotted against threshold probability for the full logistic regression model (ProbScore2), the simplified 0–6 point bedside score, and the two default strategies of treating all patients or treating no patients as high-risk. ProbScore2 provided a higher net benefit than both default strategies and the simplified score across the clinically relevant threshold range of approximately 0.10–0.50.

To evaluate the potential clinical usefulness of ProbScore2 beyond discrimination and calibration, we performed a decision curve analysis (DCA), comparing the net benefit of using ProbScore2 to guide clinical decisions against two default strategies (treating all patients as high-risk, or treating none as high-risk) across a range of threshold probabilities. Across the clinically plausible threshold range of approximately 0.10–0.50—encompassing thresholds low enough to prioritize sensitivity for triage and high enough to prioritize specificity for resource allocation—ProbScore2 provided a higher net benefit than both the treat-all and treat-none strategies, and than the simplified 0–6 point bedside score, indicating that using the model to guide decisions would be expected to identify more patients requiring closer monitoring without a proportionate increase in unnecessary interventions, compared with treating all patients uniformly. Because the prevalence of moderately severe/severe disease in this cohort was relatively high (70.2%), the advantage over the treat-all strategy was most pronounced at higher threshold probabilities; this pattern should be interpreted with the cohort’s case-mix in mind and re-examined in populations with different disease prevalence (Figure 8).

## 4. Discussion

In this single-center, retrospective cohort study of 194 patients with acute pancreatitis, we developed a new six-variable prognostic score (ProbScore2) and evaluated it through internal validation for the early identification of moderately severe/severe disease. The model was built from admission white blood cell count, blood urea nitrogen (BUN), pleural effusion, admission blood glucose level, platelet-to-lymphocyte ratio (PLR), and serum albumin. It showed excellent discriminative ability (AUC = 0.873), achieving a numerically higher AUC than all six conventional severity scores evaluated within this same retrospective, single-center patient population, including the Glasgow-Imrie score (AUC = 0.832), CTSI (0.775), the Balthazar score (0.758), BISAP (0.725), the Ranson score (0.676), and HAPS (0.574). The simplified, unweighted, 0–6 point bedside version of the score retained most of this discriminative ability (AUC = 0.852); the optimal threshold of ≥3 points yielded a sensitivity of 77.0% and a specificity of 79.7%.

The relative ranking of conventional scoring systems in our cohort was consistent with the literature, although absolute AUC values differed: Lee and Cho’s 2022 review reported AUCs of 0.810 (Ranson), 0.780 (Glasgow), and 0.875 (BISAP) [7], whereas in our population Glasgow performed better (0.832) and BISAP worse (0.725) than these pooled estimates, likely reflecting differences in case mix and single-center design. HAPS performed particularly poorly (AUC = 0.574, *p* = 0.064), most likely because none of our patients had documented peritonitis, one of its three components, consistent with limitations noted in its original validation [15]. This comparison is also not fully time-matched: Ranson, Glasgow-Imrie, Balthazar, and CTSI incorporate data collected up to 72 h after admission, whereas ProbScore2 relies exclusively on admission data. If anything, this asymmetry works against ProbScore2, which matched or exceeded these scores despite using less and earlier information. A prospective, time-matched comparison remains an important goal for future validation.

Each retained variable corresponds to a biological domain linked to disease severity: albumin to capillary leak and reduced oncotic pressure, BUN and glucose to established early markers of organ stress, PLR to systemic inflammation, and pleural effusion to extrapancreatic fluid extravasation. This may help explain why their combination outperformed scores built on a narrower physiological range [10,18,19,20,21]. Because our design is retrospective and observational, however, these associations cannot establish causal pathways. They should be read as plausible rationale from the literature rather than mechanisms demonstrated by this study. NLR, despite prior evidence of prognostic value, was excluded from the final model, most likely due to collinearity with white blood cell count and PLR [22].

An advantage of the model is that all six variables are routinely available already at admission: five come from the blood count and basic biochemistry, and the sixth (pleural effusion) requires only a plain chest radiograph rather than cross-sectional imaging. This eliminates the need for the 48-h observation period required by the Ranson or Glasgow scores, the intensive care physiological data required by APACHE II, or the contrast-enhanced CT scan required for Balthazar/CTSI grading. This directly addresses the practical problem identified in the introduction: the need for an early, within-hours post-admission predictive tool that can be used at primary or regional care level using only basic laboratory testing and a plain chest radiograph, without requiring intensive care support or advanced, CT-based imaging facilities [7].

The good agreement between the full logistic regression model and its simplified 0–6 point version (AUC: 0.873 vs. 0.852) is clinically meaningful, as it indicates that most of the prognostic information can be preserved in an unweighted form that is easy to calculate at the bedside, similar to the BISAP scoring system, without requiring electronic calculation of logistic regression coefficients. This finding is consistent with the principle that also led to the simplification of APACHE II and the development of the BISAP scoring system [13,14]. It may also facilitate broader adoption of the score in emergency and gastroenterology departments, where rapid, calculator-free risk assessment is needed to determine monitoring intensity and to decide on transfer to a tertiary center, in line with current recommendations for early risk assessment in acute pancreatitis [23].

Machine learning–based models, including risk-prediction tools such as EASY-APP [24], have also been proposed for predicting AP severity. XGBoost-based models have achieved AUCs of 0.906–0.921 [25,26], while a large multicenter model using LASSO-based variable selection with external validation demonstrated excellent discrimination for critical illness [27]. These performances are comparable to, and in some cases modestly exceed, that of ProbScore2 (AUC = 0.873). However, most machine learning approaches rely on complex black-box or ensemble algorithms that are less interpretable at the bedside, often require a larger number of input variables, and—apart from a few notable exceptions—lack external validation. In contrast, a transparent six-variable model that can be readily translated into a simple additive bedside score provides a practical balance between predictive performance and clinical usability, while preserving the interpretability clinicians need for routine risk assessment.

Baseline comparisons further supported the internal consistency of the severity classification: hypertension, SIRS, and pleural effusion were all significantly more frequent in the MODSEV group, which also had longer hospitalization and all in-hospital deaths (11.6% vs. 0%), consistent with the “multiple hits” model of metabolic syndrome components reinforcing disease severity [28]. In contrast, liver steatosis and elevated FIB-4 risk did not differ between groups, suggesting metabolic liver involvement is a common but non-discriminating comorbid finding in this population, consistent with reports that MAFLD’s relationship with AP severity is heterogeneous and dose-dependent on metabolic component burden rather than uniformly predictive [29].

This study has several limitations. First, its retrospective, single-center design carries a risk of referral and selection bias and may limit generalizability to populations with a different etiological distribution, referral pattern, or healthcare setting than our tertiary gastroenterology department. Multicenter studies across diverse settings and populations are needed before ProbScore2 is ready for widespread adoption. Second, although the sample size (*n* = 194) is comparable to many single-center model-development studies, the events-per-variable ratio for the six-variable model approaches the traditionally recommended threshold of ten [30], which could bias the regression coefficient estimates. Third, bootstrap resampling (1000 replicates) and repeated 10-fold cross-validation showed only a modest reduction in discriminative performance (AUC 0.873 apparent vs. 0.853–0.856 internally validated) and in calibration (calibration slope 1.0 apparent vs. 0.867 corrected), but both procedures resample from the same single-center cohort; per TRIPOD recommendations, prospective external validation in independent, multicenter cohorts remains essential to confirm reproducibility, calibration, and clinical applicability before routine implementation [17]. Fourth, APACHE II was not available for all patients, precluding comparison with this widely used reference scale. Fifth, pleural effusion was assessed from routine clinical chest X-ray reports rather than a dedicated, blinded reading protocol; inter-observer reliability was not formally quantified and should be addressed in future studies. Sixth, a penalized-regression (LASSO) sensitivity analysis retained only half of the same variables as our backward-stepwise model (3 of 6), reflecting some instability inherent to variable selection among correlated predictors in a moderate-sized cohort, reinforcing the need for external validation with a pre-specified variable set. Seventh, the simplified score’s cut-offs were derived and evaluated in the same cohort. Bootstrap analysis re-deriving cut-offs in each resample showed only a modest reduction in AUC (0.853 apparent vs. 0.827 corrected), but the BUN threshold was notably less stable than the others, and these fixed cut-offs should be treated as provisional pending external confirmation.

## 5. Conclusions

In this cohort of 194 patients with acute pancreatitis, a new prognostic score (ProbScore2) combining admission white blood cell count, blood urea nitrogen, pleural effusion, glucose level, platelet-to-lymphocyte ratio, and serum albumin demonstrated excellent discriminative performance (AUC = 0.873) for identifying moderately severe/severe disease, with a numerically higher AUC than the Glasgow-Imrie, CTSI, Balthazar, BISAP, Ranson evaluated within this same retrospective cohort. These comparative findings should be regarded as hypothesis-generating and require confirmation through prospective external validation before ProbScore2 is considered for clinical use. A simplified, unweighted 0–6 point bedside adaptation retained most of this discriminative ability (AUC = 0.852), with a cut-off of ≥3 points offering a clinically useful balance of sensitivity (77.0%) and specificity (79.7%).

Because five of the six components are available from routine admission blood tests and the sixth (pleural effusion) requires only a plain chest radiograph rather than cross-sectional imaging, the score can in principle be calculated within the first hours of hospitalization using resources available even in primary or regional centers without intensive care or advanced, CT-based imaging capabilities, potentially supporting earlier and more consistent decisions regarding monitoring intensity and transfer to a higher level of care. However, these findings are derived from internal validation in a single retrospective cohort, and ProbScore2 is not yet ready for routine clinical use. Before it can be adopted into clinical practice, prospective external validation in independent, multicenter cohorts is required to confirm its reproducibility, calibration, and clinical applicability; until such validation is completed, ProbScore2 should be regarded as a promising, hypothesis-generating tool rather than a validated instrument ready to guide individual patient management.

## Figures and Tables

**Table 1:** Baseline demographic, clinical, hemodynamic, nutritional, and laboratory characteristics of the study population.

Variable	MILD (*n* = 59)	MODSEV (*n* = 135)	*p* Value
**Demographic Characteristics**			
Age (years)	54.68 ± 16.32	57.30 ± 16.64	0.270
Gender (male/female), *n* (%)	37 (62.7%)/22 (37.3%)	88 (65.2%)/47 (34.8%)	0.741
**Clinical Outcomes**			
Length of hospital stay (days)	6.97 ± 1.99	9.61 ± 5.25	**<0.001**
Death, *n* (%)	0 (0%)	14 (11.57%)	**0.01**
Complications, *n* (%)			**<0.001**
No complications	20	26	
Peripancreatic fluid collection	33	49	
Pseudocyst	0	6	
Necrotic collection	3	51	
De novo diabetes	3	3	
Key Admission Predictors (ProbScore2 components; pleural effusion in Supplementary Table S1)			
WBC at admission, median (IQR) (×10^3^/µL)	11.80 (7.995–13.75)	14.10 (11.60–17.37)	**<0.001**
BUN, median (IQR) (mg/dL)	14.00 (11.00–18.57)	18.98 (13.30–27.00)	**0.001**
Blood glucose at admission, median (IQR) (mg/dL)	115.00 (99.00–142.50)	144.00 (108.00–182.50)	**0.003**
PLR at admission, median (IQR)	174.0 (107.0–218.0)	221.5 (142.2–303.0)	**0.001**
Serum albumin at admission (g/dL)	3.88 ± 0.37	3.46 ± 0.55	**<0.001**

Data are presented as mean ± SD for continuous variables and as *n* (%) for categorical variables. MILD, mild acute pancreatitis; MODSEV, moderately severe/severe acute pancreatitis. Bold *p* values indicate statistical significance (*p* < 0.05).

**Table 2:** Final logistic regression model (ENTER method).

Variable	B	S.E.	Wald	df	*p* Value	OR (95% CI)
White blood cell count at admission (per 1000 cells/µL)	0.1776	0.0519	11.708	1	<0.001	1.194 (1.079–1.322)
Blood urea nitrogen (BUN)	0.0469	0.0237	3.899	1	0.048	1.048 (1.000–1.098)
Pleural effusion	0.947	0.499	3.604	1	0.058	2.578 (0.970–6.855)
Glucose level at admission	0.0098	0.0036	7.328	1	0.007	1.010 (1.003–1.017)
Platelet-to-lymphocyte ratio (PLR) at admission (corrected)	0.0037	0.0020	3.291	1	0.070	1.004 (1.000–1.008)
Serum albumin	−2.068	0.506	16.693	1	<0.001	0.126 (0.047–0.341)
Constant	3.083	1.946	2.509	1	0.113	21.820

Abbreviations: OR, odds ratio; CI, confidence interval.

**Table 3:** ROC analysis of the six-variable ProbScore2 for predicting moderately severe/severe acute pancreatitis.

AUC	Standard Error	95% CI
0.873	0.028	0.819–0.927 (*p* < 0.001)

**Table 4:** Optimal cut-off values based on the Youden Index.

Parameter	Optimal Cut-Off	Sensitivity	1 − Specificity	Youden Index
White blood cell count at admission	>12.72 × 10^3^/µL	66.4%	28.1%	0.383
Blood urea nitrogen (BUN)	>15.0 mg/dL	61.9%	33.3%	0.286
Glucose level at admission	>140.5 mg/dL	51.5%	24.6%	0.269
Platelet-to-lymphocyte ratio (PLR)	>219	53.7%	24.6%	0.292
Serum albumin *	<3.71 g/dL	30.6%	77.2%	0.466

* Lower values indicate greater disease severity; consequently, the Youden Index was maximized in the reverse direction (albumin < cut-off). For all other variables, values above the cut-off indicated a higher risk of moderately severe/severe acute pancreatitis (MODSEV).

**Table 5:** Comparison of AUC values across scoring systems.

Score	AUC	Rank
ProbScore2 (new score)	0.873	1
Glasgow-Imrie score	0.832	2
CTSI (CT Severity Index)	0.775	3
Balthazar score	0.758	4
BISAP score	0.725	5
Ranson score	0.676	6
HAPS (Harmless Acute Pancreatitis Score)	0.574	7

**Table 6:** Distribution of the simplified score according to disease severity.

Score	MILD (*n*)	MODSEV (*n*)	Total (*n*)	MODSEV (%)
0	11	2	13	15.4%
1	20	10	30	33.3%
2	16	19	35	54.3%
3	10	40	50	80.0%
4	2	29	31	93.5%
5	0	27	27	100.0%
6	0	8	8	100.0%

**Table 7:** ROC and Youden analysis of the simplified 0–6 point score.

Cut-Off (≥)	Sensitivity	Specificity	Accuracy	Youden Index
≥1	98.5%	18.6%	74.2%	0.172
≥2	91.1%	52.5%	79.4%	0.437
≥3	77.0%	79.7%	77.8%	0.567
≥4	47.4%	96.6%	62.4%	0.440
≥5	25.9%	100%	48.5%	0.259

## Data Availability

The datasets analyzed during the current study are available from the corresponding author on reasonable request.

## Supplementary Materials

The following supporting information can be downloaded at https://www.mdpi.com/article/10.3390/medicina62091630/s1, Figure S1. boxplots of significant admission discriminators between MILD and MODSEV groups; Table S1. Baseline clinical and anamnestic characteristics of the study population; Table S2. Additional nutritional, vital sign, inflammatory, hematological, hepatic, pancreatic, and glycemic characteristics of the study population, not included among the final ProbScore2 predictors. Data are presented as mean ± SD or median (IQR) for continuous variables and as n (%) for categorical variables; Table S3. TRIPOD (Transparent Reporting of a multivariable prediction model for Individual Prognosis Or Diagnosis) checklist, indicating where each recommended reporting item is addressed in this manuscript.

## Abbreviations

The following abbreviations are used in this manuscript:

AP	Acute pancreatitis
MILD	Mild acute pancreatitis
MODSEV	Moderately severe/severe acute pancreatitis
SIRS	Systemic inflammatory response syndrome
ARDS	Acute respiratory distress syndrome
COPD	Chronic obstructive pulmonary disease
BISAP	Bedside Index for Severity in Acute Pancreatitis
HAPS	Harmless Acute Pancreatitis Score
CTSI	CT Severity Index
mCTSI	Modified CT Severity Index
BUN	Blood urea nitrogen
NLR	Neutrophil-to-lymphocyte ratio
PLR	Platelet-to-lymphocyte ratio
CRP	C-reactive protein
WBC	White blood cell count
CONUT	Controlling Nutritional Status score
FIB-4	Fibrosis-4 Index
ROC	Receiver operating characteristic
AUC	Area under the curve
OR	Odds ratio
CI	Confidence interval
ERCP	Endoscopic retrograde cholangiopancreatography
MAFLD	Metabolic-associated fatty liver disease
TRIPOD	Transparent Reporting of a Multivariable Prediction Model for Individual Prognosis or Diagnosis
