# Supplementary material for: New Perspectives in the Assessment of Severity Scoring in Acute Pancreatitis: Development and Internal Validation of a Novel Bedside Prognostic Score (ProbScore2)
Source: Medicina (Kaunas). 2026 Aug 25;62(9):1630. doi: 10.3390/medicina62091630 (PMC13608798; doi:10.3390/medicina62091630)
Supplement: Supplementary file 1 [file medicina-62-01630-s001.zip › Table S1.pdf]

Table S1. Baseline clinical and anamnestic characteristics of the study population.

Variable	MILD (n = 59)	MODSEV (n = 135)	p value
Cardiovascular Comorbidities			
Hypertension, n (%)	31 (52.5%)	92 (68.1%)	**0.038**
Chronic ischemic cardiopathy, n (%)	4 (6.8%)	22 (16.3%)	0.073
Atrial fibrillation, n (%)	2 (3.4%)	10 (7.4%)	0.285
Acute heart failure, n (%)	5 (8.5%)	17 (12.6%)	0.405
Valvular disease, n (%)	7 (11.9%)	13 (9.6%)	0.638
Acute myocardial infarction (history), n (%)	1 (1.7%)	5 (3.7%)	0.457
Deep vein thrombosis, n (%)	1 (1.7%)	9 (6.7%)	0.150
Respiratory Comorbidities			
Respiratory disease (COPD, asthma), n (%)	8 (13.6%)	20 (14.8%)	0.819
ARDS, n (%)	2 (3.4%)	16 (11.9%)	0.062
Pleural effusion, n (%)	8 (13.6%)	59 (43.7%)	**<0.001**
SIRS, n (%)	25 (43.1%)	87 (64.9%)	**0.005**
Metabolic and Renal Comorbidities			
Diabetes mellitus, n (%)	9 (15.3%)	36 (26.7%)	0.083
Chronic kidney disease, n (%)	4 (6.8%)	23 (17.0%)	0.058
Hepatic Findings			
Liver steatosis, n (%)	41 (69.5%)	110 (81.5%)	0.064
FIB-4 score (fibrosis risk)			0.501
— Low risk	18 (30.5%)	34 (25.2%)	
— Indeterminate risk	11 (18.6%)	35 (25.9%)	
— High risk	30 (50.8%)	66 (48.9%)	
Neurological and Other Comorbidities			
Stroke, n (%)	4 (6.8%)	9 (6.7%)	0.977
Osteoarticular disease, n (%)	10 (16.9%)	18 (13.3%)	0.510
Paraneoplasia, n (%)	5 (8.5%)	8 (5.9%)	0.514

Variable	MILD (n = 59)	MODSEV (n = 135)	p value
Peritonitis, n (%)	0 (0%)	0 (0%)	—
Upper GI Endoscopy Findings			
Endoscopy performed, n (%)	22 (37.3%)	36 (26.7%)	0.137
Gastritis, n (%)	16 (27.1%)	21 (15.6%)	0.059
Esophagitis, n (%)	9 (15.3%)	14 (10.4%)	0.333
Gastropathy, n (%)	3 (5.1%)	7 (5.2%)	0.977
Erosion, n (%)	4 (6.8%)	9 (6.7%)	0.977
Gastric ulcer, n (%)	2 (3.4%)	4 (3.0%)	0.874
Duodenitis, n (%)	4 (6.8%)	8 (5.9%)	0.820
Mallory-Weiss lesion, n (%)	4 (6.8%)	8 (5.9%)	0.820
Tumor at endoscopy, n (%)	3 (5.1%)	1 (0.7%)	0.050
Bleeding, n (%)	0 (0%)	1 (0.7%)	0.507
Endoscopic localization: Cardia, n (%)	4 (6.8%)	1 (0.7%)	**0.015**
Endoscopic localization: Fundus, n (%)	4 (6.8%)	0 (0%)	**0.002**
Endoscopic localization: D2, n (%)	4 (6.8%)	1 (0.7%)	**0.015**
Medication			
Proton pump inhibitor use, n (%)	44 (74.6%)	112 (83.0%)	0.176
Etiology			0.849
Biliary, n (%)	23 (39.0%)	50 (37.0%)	
Alcohol-induced, n (%)	15 (25.4%)	33 (24.4%)	
Metabolic-associated, n (%)	12 (20.3%)	35 (25.9%)	
Other, n (%)	9 (15.3%)	17 (12.6%)	
Toxic Habits			0.408
Alcohol consumption, n (%)	10 (16.9%)	20 (14.8%)	
Smoking, n (%)	8 (13.6%)	13 (9.6%)	
Both, n (%)	12 (20.3%)	43 (31.9%)	

Abbreviations: MILD, mild acute pancreatitis; MODSEV, moderately severe/severe acute pancreatitis; ARDS, acute respiratory distress syndrome; SIRS, systemic inflammatory response syndrome; COPD, chronic obstructive

pulmonary disease; FIB-4, Fibrosis-4 Index; GI, gastrointestinal. Bold p values indicate statistical significance ($p < 0.05$).
