# Supplementary material for: New Perspectives in the Assessment of Severity Scoring in Acute Pancreatitis: Development and Internal Validation of a Novel Bedside Prognostic Score (ProbScore2)
Source: Medicina (Kaunas). 2026 Aug 25;62(9):1630. doi: 10.3390/medicina62091630 (PMC13608798; doi:10.3390/medicina62091630)
Supplement: Supplementary file 1 [file medicina-62-01630-s001.zip › Table S2.pdf]

**Table S2.** Additional nutritional, vital sign, inflammatory, hematological, hepatic, pancreatic, and glycemic characteristics of the study population, not included among the final ProbScore2 predictors. Data are presented as mean ± SD or median (IQR) for continuous variables and as n (%) for categorical variables.

| Variable | MILD (n = 59) | MODSEV (n = 135) | p value |
|---|---|---|---|
| **Nutritional Assessment** | | | |
| Total cholesterol (mg/dL) | 176.32 ± 76.32 | 169.49 ± 68.51 | 0.748 |
| CONUT score | 2.73 ± 2.02 | 4.32 ± 2.90 | **0.001** |
| CONUT category, n (%) | | | **0.001** |
| — Normal | 18 (30.5%) | 20 (14.8%) | |
| — Mild malnutrition | 33 (55.9%) | 61 (45.2%) | |
| — Moderate malnutrition | 8 (13.6%) | 41 (30.4%) | |
| — Severe malnutrition | 0 (0%) | 13 (9.6%) | |
| **Vital Signs at Admission** | | | |
| Body temperature (°C) | 36.61 ± 0.48 | 36.61 ± 0.53 | 0.200 |
| Heart rate (beats/min) | 88.44 ± 15.87 | 89.96 ± 19.10 | 0.685 |
| Respiratory rate (breaths/min) | 18.56 ± 1.96 | 19.14 ± 2.99 | 0.261 |
| Systolic blood pressure (mmHg) | 140.17 ± 21.42 | 139.42 ± 24.97 | 0.892 |
| Diastolic blood pressure (mmHg) | 82.20 ± 13.18 | 81.19 ± 16.33 | 0.453 |
| Blood pressure category, n (%) | | | 0.740 |
| — Normal | 7 (11.9%) | 25 (18.5%) | |
| — Elevated | 6 (10.2%) | 12 (8.9%) | |
| — Grade I | 16 (27.1%) | 27 (20.0%) | |
| — Grade II | 17 (28.8%) | 42 (31.1%) | |
| — Grade III | 10 (16.9%) | 19 (14.1%) | |
| — Hypertensive crisis | 3 (5.1%) | 10 (7.4%) | |
| **Inflammatory Markers** | | | |
| NLR at admission, median (IQR) | 6.00 (4.00–8.28) | 11.00 (6.00–17.50) | **<0.001** |
| NLR at 24 h, median (IQR) | 4.00 (3.00–6.25) | 9.00 (5.62–17.00) | **<0.001** |
| NLR at 48 h, median (IQR) | 4.00 (2.00–5.03) | 7.60 (4.97–12.07) | **<0.001** |

| | | | |
|---|---|---|---|
| PLR at 24 h, median (IQR) | 138.5 (110.8–194.0) | 184.0 (133.7–275.0) | **0.001** |
| PLR at 48 h, median (IQR) | 131.0 (97.8–169.8) | 163.0 (124.5–232.2) | **<0.001** |
| CRP at admission, median (IQR) (mg/L) | 20.00 (6.00–82.00) | 72.50 (15.50–198.00) | **<0.001** |
| **Hematological Parameters** | | | |
| Hematocrit at admission (%) | 40.63 ± 5.78 | 41.92 ± 6.17 | 0.233 |
| Hematocrit at 24 h (%) | 38.88 ± 5.15 | 39.45 ± 6.05 | 0.624 |
| Platelet count (×10³/μL) | 222.77 ± 89.99 | 228.33 ± 89.86 | 0.785 |
| Lymphocyte count (×10³/μL) | 1427.95 ± 611.92 | 1286 ± 997.63 | **0.008** |
| **Renal Function** | | | |
| Serum creatinine, median (IQR) (mg/dL) | 0.77 (0.62–0.97) | 0.94 (0.70–1.19) | **0.001** |
| **Hepatic Function** | | | |
| AST/GOT (U/L) | 168.38 ± 239.16 | 177.64 ± 219.53 | 0.411 |
| ALT/GPT (U/L) | 137.34 ± 197.76 | 153.68 ± 204.09 | 0.454 |
| **Pancreatic Enzymes** | | | |
| Serum amylase (U/L) | 1048.12 ± 1501.69 | 1019.69 ± 1135.67 | 0.404 |
| **Glycemic Parameters** | | | |
| Peak glucose during hospitalization, median (IQR) (mg/dL) | 116.00 (99.50–147.50) | 151.00 (120.00–196.00) | **<0.001** |
