# Supplementary material for: New Perspectives in the Assessment of Severity Scoring in Acute Pancreatitis: Development and Internal Validation of a Novel Bedside Prognostic Score (ProbScore2)
Source: Medicina (Kaunas). 2026 Aug 25;62(9):1630. doi: 10.3390/medicina62091630 (PMC13608798; doi:10.3390/medicina62091630)
Supplement: Supplementary file 1 [file medicina-62-01630-s001.zip › Table S3.pdf]

Table S3. TRIPOD (Transparent Reporting of a multivariable prediction model for Individual Prognosis Or Diagnosis) checklist, indicating where each recommended reporting item is addressed in this manuscript.

Section/Item	TRIPOD Checklist Item	Reported?	Location in Manuscript
Title (1)	Identify as a model development study; specify target population and outcome	Yes	Title, p. 1
Abstract (2)	Structured summary of objectives, design, setting, participants, predictors, outcome, and results	Yes	Abstract, p. 2-4
Background (3a)	Explain medical context and rationale, including existing prediction models	Yes	Introduction, p. 5-9
Objectives (3b)	Specify study objectives, including whether development or validation	Yes	Introduction (Aim of the Study), p. 10
Source of data (4a)	Describe study design	Yes	Methods 2.1, p. 11-15
Source of data (4b)	Key study dates, including recruitment period	Yes	Methods 2.1, p. 11-15
Participants (5a)	Eligibility criteria for participants	Yes	Methods 2.1, p. 14
Participants (5b)	Details of treatments received, if relevant	N/A	Observational study; no intervention
Participants (5c)	Study recruitment dates and setting	Yes	Methods 2.1 / Figure 1, p. 11, 14
Outcome (6a)	Define outcome and how/when assessed	Yes	Methods 2.2, p. 16
Outcome (6b)	Blinding of outcome assessors	N/A	Retrospective chart review
Predictors (7a)	Define all predictors and how/when measured	Yes	Methods 2.4, p. 19–23 (incl. comparator score timing)
Predictors (7b)	Blinding of predictor assessors	N/A	Retrospective chart review

Sample size (8)	Explain how the study size was arrived at	Yes	Methods 2.1, p. 13
Missing data (9)	How missing data were handled	Yes	Methods 2.5 / Results 3.3, p. 27, p. 50
Statistical analysis (10a–c)	Relationship between predictors and outcome; model-building and variable-selection procedures; how predictors were handled	Yes	Methods 2.5 / Results 3.3, p. 24, 28, p. 50–54 (backward stepwise + LASSO sensitivity analysis)
Statistical analysis (10d)	Internal validation methods	Yes	Methods 2.5 / Results, p. 28–29, p. 63–64, p. 69 (bootstrap optimism correction, discrimination, calibration, and simplified score)
Risk groups (11)	Explain how risk groups were created	Yes	Results 3.4, p. 62–63 (0–6 point bedside score, cut-off ≥3)
Development vs. validation (12)	For validation studies, describe differences from development data	N/A	Development-only study; external validation identified as future work
Participant flow (13a)	Flow of participants through the study	Yes	Figure 1 (STROBE-style diagram), p. 14
Participant characteristics (13b)	Baseline characteristics and missing data	Yes	Table 1, p. 43–44
Model development (14a)	Number of participants/events per predictor (EPV)	Yes	Methods 2.1 / Limitations, p. 13, p. 80
Model development (14b)	Unadjusted association between each predictor and outcome	Yes	Table 1, p. 43–44
Full model (15a)	Present the full prediction model (all coefficients and	Yes	Table 2 and full model equation, p. 53–54, 55

	intercept)		
Model use (15b)	Explain how to use the model in practice	Yes	Figure 6 (clinical workflow diagram), p. 68 (introduced p. 55); simplified 0–6 point score
Model performance (16)	Report discrimination and calibration measures	Yes	Results 3.3–3.4, p. 46–73 (AUC, calibration plot/slope/intercept, Brier score, decision curve analysis)
Model performance (17)	For validation studies, compare performance to development data	N/A	Development-only study
Limitations (18)	Discuss study limitations (sample size, missing data, generalizability, etc.)	Yes	Discussion (Limitations), p. 80–82 (seven numbered limitations)
Interpretation (19b)	Overall interpretation considering objectives, limitations, and other evidence	Yes	Discussion, p. 73–79 (incl. comparison to established scores and machine-learning models)
Implications (20)	Discuss potential clinical use and implications for future research	Yes	Discussion / Conclusions, p. 82–84 (external validation needs, clinical workflow)
Supplementary information (21)	Availability of supplementary resources (protocols, data, code)	Yes	Supplementary Materials section, p. 84–85
Funding (22)	Source of funding and role of funders	Yes	Funding statement, p. 93
